# Phenotypical and biochemical characterization of murine psoriasiform and fibrotic skin disease models in Stabilin‐deficient mice

**DOI:** 10.1002/2211-5463.13857

**Published:** 2024-06-30

**Authors:** Jessica Krzistetzko, Cyrill Géraud, Christof Dormann, Anna Riedel, Thomas Leibing

**Affiliations:** ^1^ Department of Dermatology, Venereology, and Allergology, University Medical Center and Medical Faculty Mannheim Heidelberg University Mannheim Germany; ^2^ Section of Clinical and Molecular Dermatology, University Medical Center and Medical Faculty Mannheim Heidelberg University Mannheim Germany; ^3^ European Center for Angioscience (ECAS), Medical Faculty Mannheim Heidelberg University Mannheim Germany

**Keywords:** Imiquimod (IMQ), psoriasis, scleroderma, Stabilin‐1 (Stab1), Stabilin‐2 (Stab2)

## Abstract

Stabilin‐1 (Stab1) and Stabilin‐2 (Stab2) are scavenger receptors expressed by liver sinusoidal endothelial cells (LSECs). The Stabilin‐mediated scavenging function is responsible for regulating the molecular composition of circulating blood in mammals. Stab1 and Stab2 have been shown to influence fibrosis in liver and kidneys and to modulate inflammation in atherosclerosis. In this context, circulating and localized TGFBi and POSTN are differentially controlled by the Stabilins as their receptors. To assess Stab1 and Stab2 functions in inflammatory and fibrotic skin disease, topical Imiquimod (IMQ) was used to induce psoriasis‐like skin lesions in mice and Bleomycin (BLM) was applied subcutaneously to induce scleroderma‐like effects in the skin. The topical treatment with IMQ, as expected, led to psoriasis‐like changes in the skin of mice, including increased epidermal thickness and significant weight loss. Clinical severity was reduced in Stab2‐deficient compared to Stab1‐deficient mice. We did not observe differential effects in the skin of Stabilin‐deficient mice after bleomycin injection. Interestingly, treatment with IMQ led to a significant increase of Stabilin ligand TGFBi plasma levels in *Stab2* ^−/−^ mice, treatment with BLM resulted in a significant decrease in TGFBi levels in *Stab1* ^−/−^ mice. Overall, Stab1 and Stab2 deficiency resulted in minor alterations of the disease phenotypes accompanied by alterations of circulating ligands in the blood in response to the disease models. Stabilin‐mediated clearance of TGFBi was altered in these disease processes. Taken together our results suggest that Stabilin deficiency‐associated plasma alterations may interfere with preclinical disease severity and treatment responses in patients.

## Graphical abstract

Stabilin‐1 and Stabilin‐2 deficiency in mouse models of skin disease revealed altered circulating ligands but only minor alterations in disease phenotypes. Psoriasis‐like changes are reduced in Stab2‐deficient mice with increased TGFBi levels compared to Stab1‐deficient mice, while scleroderma‐like effects show no differential impact. These findings highlight Stabilins' role in modulating disease severity and circulating ligand dynamics, potentially impacting treatment strategies.

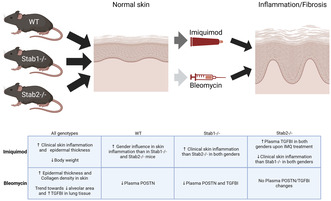

**Abbreviations**
DAPI: 4′,6‐diamidino‐2‐phenylindole
ECAS: European Center for Angioscience
ELISA: Enzyme‐Linked Immunosorbent Assay
H&E: Hematoxylin & Eosin
HIER: heat‐induced epitope retrieval
IF: immunofluorescence
IMQ: Imiquimod
JMP: statistical software (by SAS Institute)
PAS: periodic acid‐schiff (staining method)
PASI: psoriasis area and severity index
PBS: phosphate buffered saline
Postn: periostin
Sirius Red: dye used in staining for collagen
Stab1: Stabilin‐1
Stab2: Stabilin‐2
TECAn: Thermo Fisher Scientific
TGFBi: transforming growth factor beta‐induced
WT: wildtype

Approximately 2–5% of the world's population suffers from psoriasis [1, 2]. Psoriasis is a chronic inflammatory skin disease histologically characterized by epidermal hyperplasia (acanthosis), epidermal leukocyte infiltration (parakeratosis), marked keratinocyte hyperproliferation, inflammation and increased vascularity of the skin [3, 4, 5, 6]. This immune‐mediated condition is underpinned by dysregulation of immune cells and keratinocytes but the pathogenesis is not fully understood at present [7].

It is well known that there is an association between psoriasis and non‐alcoholic fatty liver disease (NAFLD) in humans [8, 9]. A few studies have demonstrated that study participants with psoriasis have a higher risk of NAFLD [8, 9, 10]. Among examined participants with NAFLD, the rate of psoriasis ranged from approximately 42–46% [8, 10]. Another study indicated that participants with psoriasis have a twofold higher risk of NAFLD compared to participants without psoriasis [9].

Scleroderma is a highly complex disorder with a variety of clinical manifestations. It involves a disruption of connective tissue, typically manifesting as thickening and hardening of the skin. In addition to these symptoms, some patients experience swollen fingers, musculoskeletal inflammation, severe fatigue and vascular dysfunction [11, 12, 13]. The factors influencing the development of scleroderma are not fully understood at present. During the disease, there is an activation of the immune system, leading to blood vessel and tissue damage, resulting in the formation of scar tissue and accumulation of excess collagen [14].

Endothelial cells (ECs) of the liver, the so‐called liver sinusoidal endothelial cells (LSECs) are characterized by their fenestrations between the ECs, missing diaphragms and a basal membrane. These properties make LSECs among the most permeable ECs in the body of mammals [15, 16, 17]. One of the tasks of LSECs is the high capacity to uptake soluble macromolecules and nanoparticles from the blood, for example viruses. In order to fulfill this task, LSECs express a variety of different endocytosis receptors, including the Scavenger receptors (SRs) Stabilin‐1 (Stab1) and Stabilin‐2 (Stab2) [18, 19, 20, 21]. SRs are defined as cell surface receptors that typically bind a variety of ligands and are responsible for the clearance of modified substances. They are involved in various cellular processes such as endocytosis, phagocytosis, adhesion and signaling which result in the elimination of degraded or hazardous substances [22].

Stab1 and Stab2 are large type‐1 transmembrane proteins that belong to the class H of SRs [21, 23]. Both Stabilins possess 7 Fasciclin domains as well as multiple Epidermal Growth Factor (EGF) domains [21], through which they bind ligands such as AGEs, bacteria or various glycosaminoglycans, as well as one hyaluronan (HA) binding link domain, which is non‐functional in Stab1 [21, 24, 25, 26, 27].

The 280 kDa large Stab1, also known as FEEL‐1 (Fasciclin, EGF‐like, laminin‐type EGF‐like and link domain‐containing scavenger receptor‐1) and CLEVER‐1 (common lymphatic endothelial and vascular endothelial receptor‐1) is constitutively expressed by sinusoidal ECs (liver, spleen, lymph nodes and bone marrow) and a subset of macrophages [21, 23, 24, 28, 29, 30]. The 275 kDa large Stab2, which also goes by the names HARE (hyaluronan receptor for endocytosis) due to its ability to bind hyaluronan, and FEEL‐2 (Fasciclin, EGF‐like, laminin‐type EGF‐like and link domain‐containing scavenger receptor‐2), is similarly expressed by sinusoidal ECs and in contrast to Stab1 absent from macrophages in mice [31, 32, 33].

In previous studies, our group already demonstrated that Stab1 and Stab2 exhibit different mechanisms of action in various organs and diseases. It has been shown that the combined knockout of Stab1 and Stab2 (*Stab1*/*Stab2* ^−/−^) leads to severe renal glomerulofibrosis, albuminuria mild perisinusoidal liver fibrosis and premature death of mice [34]. Seven weeks after the transplantation of double‐knockout (DKO) kidneys into wildtype (WT) mice, renal glomerulofibrosis showed a significant improvement in comparison to DKO mice. This finding suggests that the Stab1 and Stab2 mediated clearance function in LSECs is necessary for physiological homeostasis in distant organs, too [34].

Furthermore, it has been demonstrated that the single deficiency of Stab1 or Stab2 (via genetic knockout or anti‐Stabilin‐antibody therapy) significantly reduced plaque formation in the aorta in *ApoE* ^−/−^ and *Ldlr* ^−/−^ models, likely through immunomodulatory effects through an altered plasma proteome [33]. We also could show that there is a strong correlation between liver fibrosis and the abundance of Stabilin ligand Transforming growth factor beta‐induced (TGFBi) [35] and Stabilin ligands are deposited in enlarged glomeruli in an age‐dependent manner [36].

Transforming growth factor beta‐induced (TGFBi) and Periostin (Postn) are secreted matricellular proteins that consist of four fasciclin domains and one EMI domain [37, 38]. TGFBi and Postn are direct Stabilin ligands (likely due to fasciclin domain binding) and belong, same as Stabilins, to the family of Fasciclin domain proteins. Together the four proteins represent the only four Fasciclin domain proteins in mammals. Human plasma proteome profiling revealed an increase in both TGFBi and Postn in aging [39]. In human patients, single nucleotide polymorphisms in Stab1 or Stab2 induced pleiotropic plasma proteome changes, including Postn [40].

It has already been demonstrated that TGFBi is expressed in human and murine skin [41, 42]. Immunofluorescence analyses show that TGFBi is predominantly expressed in the region around the basement membrane and is localized subepidermally [43]. Furthermore, it has been shown that TGFBi is involved in wound healing in addition to cell growth and apoptosis [43, 44]. The literature has scarcely described the association of TGFBi with inflammatory and fibrotic skin diseases.

Postn has a role in chronic inflammation and fibrosis of various tissues and its involvement in the development of bones, teeth, and cartilage [38, 45, 46]. Various studies have already demonstrated that Postn is expressed in human and murine skin and is required for the normal development and homeostasis of the skin [47, 48]. The literature also describes the role of Postn in various skin diseases and wound healing, including psoriasis and scleroderma [47, 49]. It is described that the absence of Postn in chronically inflamed tissue correlated with an increase in inflammatory cell infiltrations [47].

To mimic psoriasis in mice the most well‐known and commonly used model is the topical application of Aldara cream which contains the agent Imiquimod (IMQ) [50]. IMQ is a ligand for Toll‐like receptors 7 and 8, recognized for inducing a psoriasis‐like dermatitis [51, 52]. A recent study could show an association between psoriasis‐like dermatitis induced by IMQ and NASH. Presence of NASH had a significant impact on the degree of psoriatic skin changes in mice. In this context, proinflammatory cytokines such as IL23a and Il1b were significantly upregulated in psoriatic lesions when NASH was concurrently present [53].

To assess the role of Stabilin deficiency in skin fibrosis as an addition to the inflammatory IMQ model, we used the subcutaneous injection of Bleomycin (BLM) to induce scleroderma‐like symptoms in mice. In mouse models the subcutaneous injection of BLM, in addition to scleroderma‐like symptoms, also leads to lung fibrosis [54, 55].

The aim of this study was to investigate the role of the Stabilins in the inflammatory and pro‐fibrotic skin disease models and their effect on the Stabilin ligands TGFBi and Postn.

## Materials and methods

### Animal studies

To investigate the influence of Stab1 and Stab2 on psoriasiform skin inflammation in mice, male and female mice deficient for either Stab1 (*Stab1* ^−/−^) *or Stab2* (*Stab2* ^−/−^) and Wildtype mice (WT) were treated with Aldara™ Cream (Meda, Sweden) with the agent Imiquimod 5% on their shaved back for 4 days each day. One application includes approximately 62.5 mg of cream which correlates with 3125 mg of the agent Imiquimod. Vehicle‐treated mice were treated with Vaseline (Linola™ Fett, Dr. August Wolff GmbH & Co. KG Arzneimittel, Bielefeld, Germany). On day 2 and 4, mice were treated with 250 μL PBS injections intraperitoneally. On day 5, mice were sacrificed.

To investigate the influence of Stab1 and Stab2 on skin fibrosis, mice were treated with Bleomycin (Sigma Aldrich, St. Louis, MO, USA). Male mice of equal genotypes (WT, *Stab1* ^−/−^ and *Stab2* ^−/−^) were subcutaneously injected daily with 100 μL Bleomycin in PBS (which corresponds to 0.1 U per injection and 0.6 mg·mL^−1^ Bleomycin per injection) on two positions (every day the same two positions) for 10 days. On day 7 no injections took place. Vehicle‐treated mice were subcutaneously injected with 100 μL PBS. On day 11 mice were sacrificed by cervical dislocation. The animal study protocol was approved by local authorities (Regierungspräsidium Karlsruhe; G144/19) and approval was granted in advance.

### Histopathological analysis

For Hematoxylin & Eosin (H&E), Sirius Red, Masson Trichrome and PAS staining formalin‐fixed, paraffin‐embedded samples were processed according to standard protocols.

### Immunofluorescence stainings

Paraffin sections (3–4 μm) were baked for 1 h at 60 °C and after that rehydrated in gradient alcohol series. The epitopes of the tissue were exposed by antigen retrieval with HIER (Heat‐induced epitope retrieval) buffer (pH 9.0) at 95 °C water bath for 30 min. Primary antibody (Table 1) was diluted in antibody diluent (Dako) and was incubated over night at 4 °C. Every antibody was diluted according to the manufacturer's specification. On day two the sections were washed in PBS and after that secondary antibody was diluted 1 : 400 in combination with DAPI, which was diluted 1 : 1000, in antibody dilution (Dako Agilent, Santa Clara, CA, USA). The sections were incubated for 1 h at room temperature.

**Table 1:** Antibody list.

Target	Vendor, order number
TGFBi	Abcam, ab170874, Cambridge, UK
Postn	BioTechne, AF2955, Minneapolis, MN , USA

### Image editing and quantification

Brightfield images (H&E, Masson, Trichrome, PAS and Sirius Red) were edited by using the image editing software fiji (56).

To quantify Sirius Red staining, the images captured under the microscope were opened in fiji (56) and split into individual channels (red, green, and blue). Using the threshold function (with Renyi Entropy mode), the stained area was marked and adjusted in the desired channel containing the color to be quantified and subsequently measured using the measure function.

To quantify collagen fibers from Masson Trichrome staining, small dermis segments were cut, channel separation was performed and the green channel area was quantified, like described above.

Immunofluorescence (IF) stains were initially processed directly after microscopy using the nis‐elements advanced research 7.30 software (Nikon, Tokyo, Japan), where the background was reduced using the Rolling Ball Substraction function. Quantification of IF stains was also performed using the image editing software fiji imagej. For this purpose, the stains were quantified based on the percentage of stained area of total area using the threshold function (with Renyi Entropy mode) and then presented in the graphs shown.

### ELISA assay

For both ELISA assays (TGFBi and Postn, Table 2) Plasma was used. Both ELISAs were performed to the manufacturer's specification. For TGFBi ELISA, Plasma was diluted 1 : 500 and for Postn ELISA, Plasma was diluted 1 : 2000. The optical density was measured with a TECAN Microplate‐Reader at the wavelength of 450 nm.

**Table 2:** ELISA list.

Kit	Target	Vendor, order number
Mouse BIGH3 (TGFBi) ELISA Kit	TGFBi	ThermoFisher Scientific, EMTGFBI, Waltham, MA, USA
Mouse Postn/OSF‐2 ELISA Kit	Postn	BioTechne, MOSF20

### PASI score

For the detection of severity of skin inflammation of Imiquimod‐treated mice a PASI‐like Score (Psoriasis Area and Severity Index) was performed. For that the skin of every mouse was photographed every day. After that every picture was blinded for treatment, day of treatment, genotype and sex. Five colleagues of the institute (including dermatologists, biologists and technical assistants) of Dermatology, Venerology and Allergology of the university hospital of Mannheim scored the blinded pictures for scaling, thickness and erythema from 0 to 4 (thereby 0 meant no changes and 4 strongest change and inflammation of skin). After that the average of all numbers was calculated.

### Statistics

The results of the quantification of the stainings were analyzed with the software graph pad prism, Version 9 (Dotmatics, Boston, MA, USA. Two separated Two‐way‐Anovas were performed, to compare either all genotypes within one treatment or to compare two treatments of one genotype. To show significances, a Šidák‐correction was performed. *Show significances between treatments and # show significances between genotypes. The symbols show the following significances: *P* ≤ 0.05 = */#; *P* < 0.01 = **/##; *P* < 0.001 = ***/###; *P* < 0.0001 = ****/####; ns means not significant. PASI scores were analyzed with the software jmp© using the Least Squares Method with emphasis on Effect leverage. Effects of Treatment, genotype, Timepoint and a full factorial analysis of Age*Genotype*Timepoint was performed.

## Results

### Topical treatment with the agent imiquimod (IMQ) leads to severe weight loss as well as psoriasis‐like skin inflammation

**Fig. 1:**
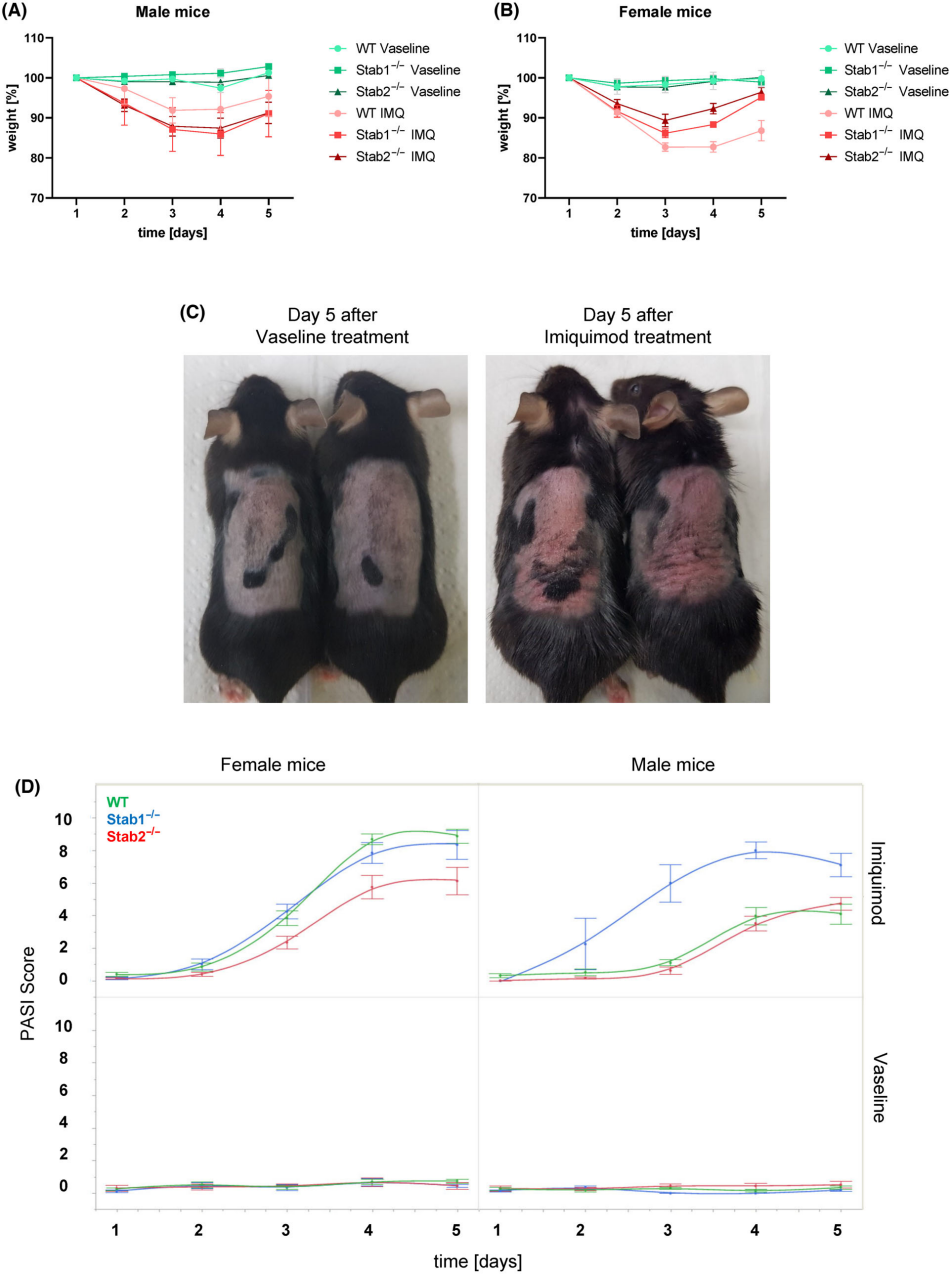
Imiquimod treatment induces psoriasiform skin inflammation. Body weight curves for (A) male (*n* = 12 for WT mice, *n* = 8 for Stab1^−/−^ and Stab2^−/−^ in each experimental condition) and (B) female (*n* = 12 for WT mice, *n* = 8 for Stab1^−/−^ and Stab2^−/−^ in each experimental condition) mice, normalized to day 1 (100%). (C) Macroscopic picture of IMQ‐treated (right) vs. Vaseline‐treated (left) WT mice. (D) PASI score (thickness, erythema, and scaling) in different experimental groups (*n* = 12 for WT mice, *n* = 8 for Stab1^−/−^ and Stab2^−/−^ in each experimental condition). Data of (A), (B), and (D) are presented as mean ± SD. To examine the influence of various variables on the weight, three‐factor analyses of variance with repeated measures were conducted. Due to numerous interactions, two separate analyses of variance were performed according to the type of feeding and treatment. The statistical analysis of the PASI score was conducted similarly to the weight analysis using a three‐factor analysis of variance.

Topical application of IMQ on the shaved back skin led to significant weight loss in all mice (Fig. 1A,B). Weight loss was noticeable from the second day onwards, but the response to the agent varied slightly between the genders and genotypes: In male mice, weight loss was not significantly altered between genotypes (Fig. 1A), while in female mice WT mice showed a significantly stronger weight loss over time (Fig. 1B).

Macroscopically, it was evident that IMQ treatment caused severe alteration of the skin (Fig. 1C and Fig. S1). The treatment with IMQ‐induced intense skin redness and scaling. Additionally, the skin became extremely hardened and wrinkling was apparent. To check the severity of macroscopic skin changes, a modified PASI Score (Psoriasis Area and Severity Index) was applied. The PASI scoring demonstrated that the genotypes of both genders responded differently to IMQ (Fig. 1D). Female *Stab2* ^−/−^ mice showed lower PASI scores compared to WT mice, while male *Stab1* ^−/−^ mice exhibited a higher PASI score than WT mice. Upon a separate examination of the PASI scoring parameters thickness, redness and scaling it was observed that female *Stab2* ^−/−^ mice presented less thickness and erythema and male *Stab1* ^−/−^ showed significantly higher scores in each category compared to respective WT cohorts (Fig. S2).

### Histological analyses of IMQ‐treated skin showed severe epidermal thickness

**Fig. 2:**
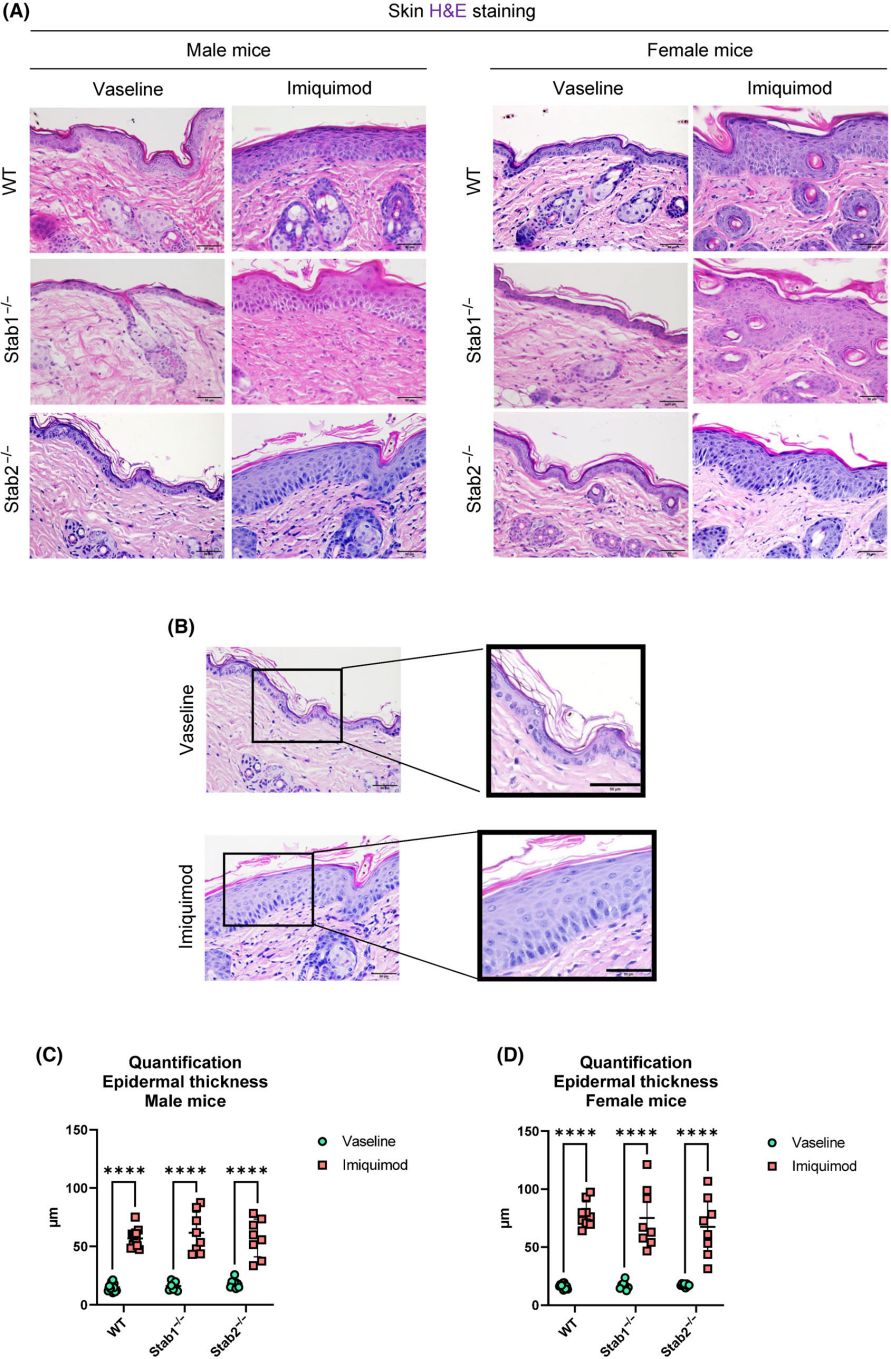
Histological analysis of mouse skin after Imiquimod treatment. (A) H&E staining of male and female skin treated with Vaseline and Imiquimod. Scale bar = 50 μm. (B) Enlarged areas of epidermis of Vaseline‐ and Imiquimod‐treated skin of female WT mice. (C) Quantification of epidermal thickness of male mice (*n* = 12 for WT mice, *n* = 8 for Stab1^−/−^ and Stab2^−/−^ in each experimental condition). (D) Quantification of epidermal thickness of female mice (*n* = 12 for WT mice, *n* = 8 for Stab1^−/−^ and Stab2^−/−^ in each experimental condition). Data of (C) and (D) are presented as mean ± SD. The statistical analysis was performed with a two‐way ANOVA. * Show significances between treatments and # show significances between genotypes. Symbols indicate significances as follows: *P* ≤ 0.05 = */#; *P* < 0.01 = **/##; *P* < 0.001 = ***/###; *P* < 0.0001 = ****/####; n.s., not significant.

Histological analysis of H&E‐stained IMQ‐treated skin sections showed severe acanthosis and mild spongiosis. In some sections, parakeratosis with occasional neutrophilic granulocytes was observed (Fig. 2A). Furthermore, IMQ led to increased epidermal thickness in all mice (Fig. 2B). The quantification of epidermal thickness showed no differences between the genotypes within both sexes comparing IMQ‐treated groups only. In female mice, there was a trend towards increased epidermal thickness compared to male mice. No significance was found between genotypes (Fig. 2C,D).

### Histological analyses of bleomycin (BLM) treated skin showed strong toxic effects and alterations in the dermis

Histological analysis of H&E stained BLM‐treated skin sections revealed that the subcutaneous injection of BLM led to pronounced toxic effects in the skin in all genotypes (Fig. S3A). Repeated injections with BLM‐induced necrotic keratinocytes and severe inflammatory reactions in form of crusts with infiltrates of neutrophilic granulocytes as well as a rarefication of hair follicles. There was a thickening of the stratum corneum and in some cases parakeratosis with spongiosis in the BLM‐treated mice.

After BLM treatment the epidermal thickness showed a significant and severe increase in all genotypes (Fig. S3B). However, the dermal thickness showed a minor and non‐significant increase in all three genotypes after BLM treatment (Fig. S3C). Between the genotypes, there were no observable differences in dermal or epidermal thickness within the respective treatment groups.

Histological analysis of Masson Trichrome stained BLM skin sections showed a severe thickening of collagen fibers and resulting in more dense connective tissue. Furthermore, Masson Trichrome staining confirmed a drastic loss of hair follicles due to BLM treatment as well (Fig. S3D–F).

### Effects of IMQ and BLM on other organs, as well as on ligands TGFBi and Postn

Since BLM induced lung fibrosis in some reports, we checked the impact on the lung in our model and observed mild structural changes (Fig. S4A–D). The histological analysis of H&E staining of lung sections revealed that subcutaneous BLM injection altered the morphology of the alveoli, resulting in decreased alveolar area across all genotypes (Fig. S4A). Alveolar area was quantified from H&E staining and showed a slight decrease in all genotypes, which was only significant in WT mice (Fig. S4C). BLM did not lead to increased collagen depositions in the lung (Fig. S4B,D).

PAS staining of kidney sections showed no abnormalities or differences, neither between the genotypes nor between the two treatment groups (Fig. S5A). Sirius Red staining of kidney sections revealed increased collagen levels in all *Stab2* ^−/−^ glomeruli with no apparent effect after BLM treatment (Fig. S5B,C).

IF stainings of the ligands TGFBi and Postn in different organs showed a significant increase in TGFBi signal in the lung of BLM‐treated WT and *Stab1* ^−/−^ mice, but not in *Stab2* ^−/−^ mice (Fig. S6A,B). Postn IF staining of lung showed no obvious influence of BLM in all genotypes (Fig. S6C,D).

In the kidneys of IMQ‐ and BLM‐treated mice, some TGFBi signal was observed in *Stab2* ^−/−^ glomeruli, which didn't differ between vehicle‐treated mice and IMQ‐ or BLM‐treated mice. Otherwise there were no differences in TGFBi and Postn signal in both treatments (Figs S7–S9).

IF staining of IMQ‐treated liver sections showed elevated TGFBi levels in male *Stab1* ^−/−^ mice, which were previously shown to correlate with liver fibrosis (Fig. S10A,B). Changes in female mice between genotypes was not significant (Fig. S10C,D).

Postn IF staining of liver sections showed no specific signal (Fig. S11).

In the BLM model, IF staining of TGFBi from liver sections of vehicle‐treated mice showed a slight trend towards higher TGFBi signal in *Stab1* ^−/−^ mice compared to WT and *Stab2* ^−/−^ mice. After BLM application, TGFBi levels in the liver were highest in *Stab1* ^−/−^ mice, while WT showed intermediate and *Stab2* ^−/−^ mice showed the lowest levels without an obvious increase in comparison to vehicle‐treated *Stab2* ^−/−^ mice (Fig. S12A,B).

IF staining of Postn from liver sections showed no specific signal (Fig. S12C,D).

### Effects of IMQ on stabilin ligands TGFBi and Postn in skin and plasma

**Fig. 3:**
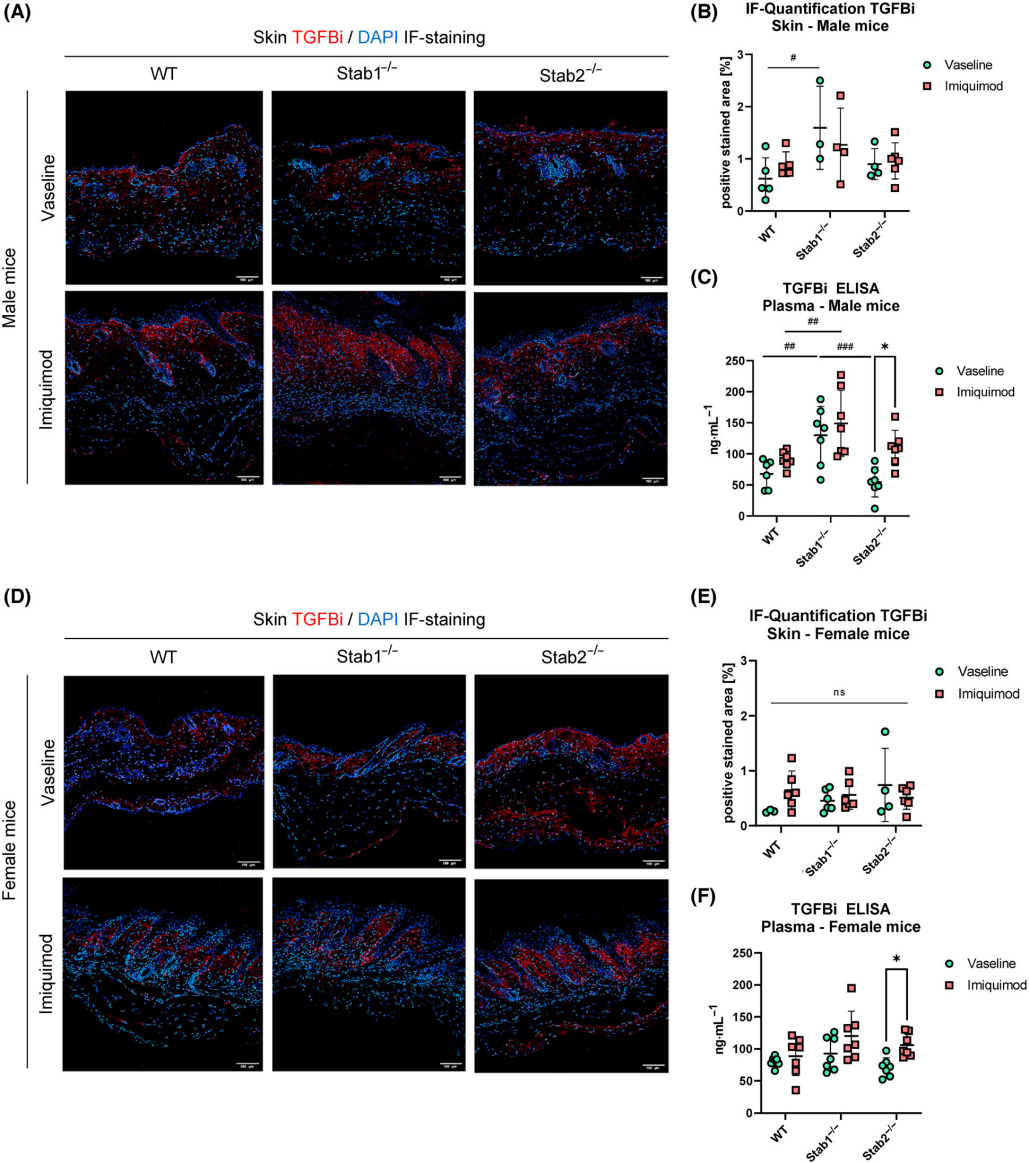
Effects of Imiquimod on stabilin ligand TGFBi in skin and plasma. (A) IF staining of TGFBi of representative skin sections in male mice. Scale bar = 100 μm. (B) Quantification of TGFBi IF staining from male mice (*n* ≥ 3). (C) Quantification of TGFBi level in plasma of male mice (*n* ≥ 7). (D) IF staining of TGFBi of representative skin sections in female mice. Scale bar = 100 μm. (E) Quantification of TGFBi IF staining from female mice (*n* ≥ 3). (F) Quantification of Postn level in plasma of female mice (*n* ≥ 7). Data of (B), (C), (E), and (F) are presented as mean ± SD. The statistical analysis was performed with a two‐way ANOVA. Symbols indicate significances as follows: *P* ≤ 0.05 = */#; *P* < 0.01 = **/##; *P* < 0.001 = ***/###; *P* < 0.0001 = ****/####; n.s., not significant.

IF staining of TGFBi in skin sections revealed positive signal in the superficial dermis. In male mice, vehicle‐treated mice of different genotypes displayed highest TGFBi levels in the skin in *Stab1* ^−/−^ mice, which was significant in comparison to WT mice (Fig. 3A,B). TGFBi plasma levels in male mice showed differences between genotypes and treatments (Fig. 3C): Vehicle‐treated *Stab1* ^−/−^ mice showed significantly higher plasma TGFBi levels than vehicle‐treated WT and *Stab2* ^−/−^ mice, which is consistent with previous findings [35, 36]. Comparing the treatments, IMQ treatment led to a significant increase in plasma TGFBi level only in *Stab2* ^−/−^ mice.

In female mice, IF staining of TGFBi showed no significant changes in the skin, only a trend towards slightly increased TGFBi signal after IMQ in WT mice was observed (Fig. 3D,E).

Plasma TGFBi levels in female mice showed a significant increase after IMQ treatment only in *Stab2* ^−/−^ mice, which is consistent with findings in male mice (Fig. 3F).

Comparing TGFBi plasma levels between sexes, male *Stab1* ^−/−^ mice showed higher TGFBi signal than female *Stab1* ^−/−^ mice (Fig. S13A). In IMQ‐treated mice, there was only a slight trend between male and female *Stab1* ^−/−^ mice, but without significance (Fig. S13B).

**Fig. 4:**
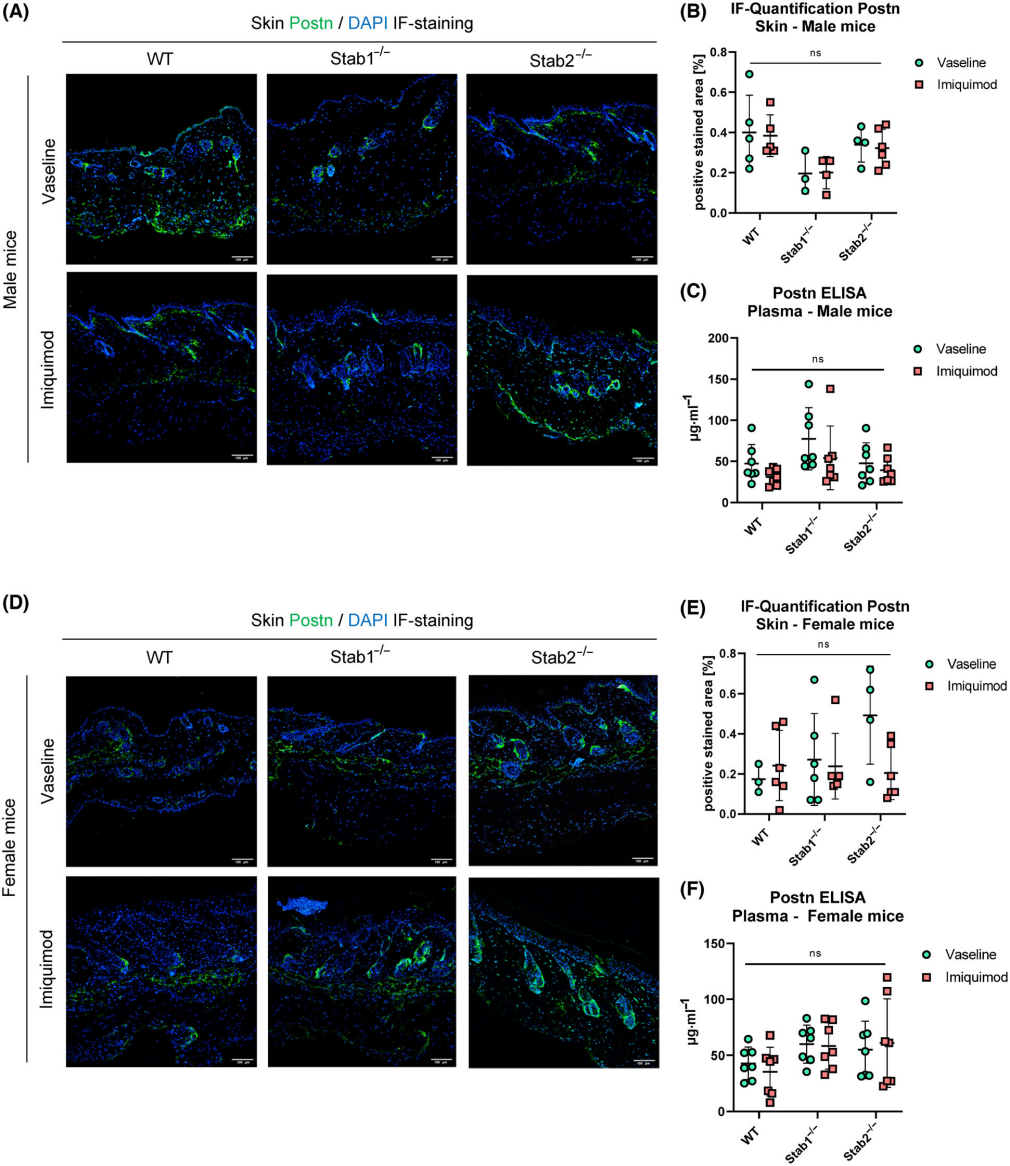
Effects of Imiquimod on stabilin ligand Postn in skin and plasma. (A) IF staining of Postn of representative skin sections in male mice. Scale bar = 100 μm. (B) Quantification of Postn IF staining from male mice (*n* ≥ 3). (C) Quantification of Postn level in plasma of male mice (*n* ≥ 7). (D) IF staining of Postn of representative skin sections in female mice. Scale bar = 100 μm. (E) Quantification of Postn IF staining from female mice. (F) Quantification of Postn level in plasma of female mice (*n* ≥ 7). Data of (B), (C), (E), and (F) are presented as mean ± SD. The statistical analysis was performed with a two‐way ANOVA. n.s., not significant.

Postn IF staining in male vehicle‐treated mice and IMQ‐treated mice of all genotypes, showed a signal below the epidermis and also in the deeper dermis, as well as around the hair follicles (Fig. 4A). Quantification of Postn staining revealed no significant differences and treatment did not induce overt changes (Fig. 4B).

Plasma Postn levels in male mice also showed no significant changes, with a trend towards higher plasma levels in *Stab1* ^−/−^ mice in both treatments (Fig. 4C).

Postn IF staining of female mice showed no significant changes between genotypes or treatments (Fig. 4D,E), which was also true for plasma Postn levels (Fig. 4F).

No differences regarding Plasma Postn between male and female mice was observed (Fig. S13C,D).

### Effects of BLM on stabilin ligands TGFBi and Postn in skin and plasma

IF staining of TGFBi from skin sections exhibited a similar signal distribution compared to the IMQ cohort (Fig. S14A). The quantification of TGFBi IF staining showed no differences of TGFBi signal between the genotypes of the vehicle‐treated group, with a trend towards higher levels in *Stab1* ^−/−^ *mice*. BLM treatment resulted in an increased TGFBi signal in the skin of WT mice, with a trend towards higher levels in *Stab1* ^−/−^ mice. BLM treatment had no obvious impact on TGFBi signal in the skin of *Stab2* ^−/−^ mice. Comparing genotypes after BLM treatment, *Stab1* ^−/−^ mice displayed significantly more TGFBi signal in the skin than *Stab2* ^−/−^ mice (Fig. S14B).

Vehicle‐treated *Stab1* ^−/−^ mice showed higher TGFBi plasma levels compared to WT and *Stab2* ^−/−^ mice, in the BLM‐treated group, significantly reduced TGFBi plasma levels in *Stab1* ^−/−^ mice were observed (Fig. S14C).

Postn IF staining from skin sections of vehicle‐treated mice predominantly showed a signal in the deeper dermis and around the hair follicles. After BLM treatment the Postn signal was visible only directly beneath the epidermis (Fig. S14D). Quantification of Postn IF staining showed no differences between vehicle‐treated mice and BLM‐treated mice and also no differences between the genotypes (Fig. S14E).

The Postn ELISA from plasma showed alterations between the treatments and genotypes. In vehicle‐treated mice, plasma Postn level was significantly higher in *Stab1* ^−/−^ mice compared to WT and *Stab2* ^−/−^ mice. After BLM treatment Postn level decreased in all genotypes, especially a very strong and significant decrease was observed in WT and *Stab1* ^−/−^ mice. In *Stab2* ^−/−^ mice the decrease was only slight and not significant (Fig. S14F).

## Discussion

In this study, we demonstrated that deficiency for Scavenger receptors Stab1 or Stab2 had some sex‐specific phenotypic effects on the response in the IMQ‐induced model of psoriasiform inflammation, but only minor effects on the BLM‐induced skin fibrosis. However, in both models, alterations of plasma levels of Stabilin ligands TGFBi and Postn were found that correlated with Stabilin deficiency. These alterations of circulating ligands were not accompanied by overt local deposition of Stabilin ligands, indicating that circulating, but not local stabilin ligands may be able to exert modulating functions in these models.

As expected, the topical application of IMQ led to the induction of a dermatitis with some similarities to human psoriasis. Mice treated with IMQ exhibited macroscopic features resembling psoriasis form inflammation like redness, scaling, skin thickening as well as histological features of psoriasis such as acanthosis and parakeratosis [3, 7, 57]. Weight loss during the IMQ treatment is also a known effect of this treatment [58, 59].

Interestingly, we found a difference in the intensity of the response to IMQ between female and male mice in different genotypes: Both weight loss and PASI scores were generally more pronounced in females compared to males. *Stab1* ^−/−^ mice showed a comparable weight loss to *Stab2* ^−/−^ in males and females, with a higher PASI found in *Stab1* ^−/−^ mice of both genders compared to *Stab2* ^−/−^. WT mice showed the highest relative weight loss in the male cohort and the lowest relative weight loss in female cohort, which correlated with a higher PASI in female WT mice and a lower PASI in WT male mice. To sum up, *Stab2* ^−/−^ mice seemed to be protected in comparison to *Stab1* ^−/−^ mice.

A potential explanation for the observed gender differences, especially in IMQ‐treated WT mice, might lie in the variation of immune responses between genders. It is known that substantial differences in immune response occur in humans depending on gender. For instance, around 80% of autoimmune diseases occur in women, who also exhibit a stronger innate and adaptive immune response than men. This circumstance includes faster pathogen clearance and greater vaccine efficacy in women compared to men, but it comes with the disadvantage of higher susceptibility to inflammatory and autoimmune diseases in women. Moreover, the stronger immune response (both innate and adaptive) of the female gender is also observed in other species such as insects, reptiles, birds, and other mammals [60].

There could be various explanations for the stronger response to IMQ in *Stab1* ^−/−^ mice compared to *Stab2* ^−/−^ mice. It is known that Stab1 is expressed in macrophages and the absence of Stab1 leads to a modulation of the immune response, resulting in increased expression of the chemokine CCL3 [61]. One reason for the stronger response in *Stab1* ^−/−^ mice could therefore be an enhanced immune response compared to *Stab2* ^−/−^ mice.

A possible explanation for the less pronounced clinical response in *Stab2* ^−/−^ mice could be higher plasmatic hyaluronic acid (HA) levels. Because Stab2 is the major receptor for HA [31], in *Stab2* ^−/−^ mice HA levels are higher than in *Stab1* ^−/−^ mice [34]. HA is a biological active glycosaminoglycan‐biopolymer which is mainly present in the extracellular matrix and has the potential to modulate inflammatory responses by regulating macrophage activation via cell surface HA receptors, like Toll‐like receptors 2 and 4 and is involved in tissue regeneration [62, 63]. In literature, it is described that transcutaneously administered hyaluronic acid nanoparticles (HA‐NP) show therapeutic efficacy against psoriasis‐like skin dermatitis by suppressing innate immune response and restoring skin barrier function without overt toxicity signs [62]. Through higher HA levels, *Stab2* ^−/−^ mice could thereby be protected from psoriasiform skin changes in our model.

Furthermore, there may be a connection between the reduced response to IMQ in *Stab2* ^−/−^ mice and elevated plasma TGFBi levels which we observed in our models. TGFBi possesses a cell‐adhesive RGD domain at its C‐terminus, consisting of the three amino acids Arginine, Glycine, and Aspartate, which serves as a ligand recognition site for various integrins [64, 65]. Integrins are a class of transmembrane cell‐adhesion molecules that are expressed on the cell surface of T‐cells, other leukocytes and most other cells in the body. Integrin signaling contributes to T‐cell circulation through peripheral lymph nodes and their migration to the center of inflammation and the retention of the T‐cells at these sites [66]. It is known that integrins play an important role in psoriasis. For example the humanized anti‐αL IgG1 monoclonal antibody Efalizumab, which causes reduced epidermal and dermal T‐cell counts and increased circulating lymphocyte counts, showed in a phase III study in patients with moderate to severe psoriasis a reduced psoriasis area and severity [66]. Thereby, integrin binding through TGFBi could possibly reduce the inflammatory response in *Stab2* ^−/−^ mice. In our previous work, we were able to show that single Stabilin‐deficiency modulates immune response in atherosclerosis, which, like psoriasis, is driven by inflammation [33]. In the inflammatory IMQ model, immunofluorescence staining of the skin indicated a slightly higher deposition of TGFBi in males (especially *Stab1* ^−/−^ mice) than in females. This was observed in both control and treated mice. This finding might suggest that TGFBi is more heavily deposited in males than in females. Significantly elevated TGFBi levels in the plasma of *Stab2* ^−/−^ mice might occur due to insufficient scavenging of Stab1 in this model. Contrarily, loss of Stab1 did not seem to influence TGFBi plasma levels in the same model, indicating a more important role for Stab2 in TGFBi binding. Alternatively, treatment with IMQ might also lead to an inhibition of Stab1 scavenging function or a combination of effects.

Postn plays a role in various skin disorders and wound healing, including psoriasis and scleroderma [47, 49]. In this study, IF staining of Postn in the skin of IMQ‐treated mice showed no differences compared to the control mice and plasma Postn levels remained unchanged after IMQ treatment in our study.

The subcutaneous injection of BLM led to mild skin sclerosis and significant toxic effects in the organs of the mice shown here. Some mice exhibited strong inflammatory skin reactions macroscopically and microscopically, and nearly all mice showed thickening of the epidermis. BLM treatment resulted in dermal thickening and increased collagen fiber density in the dermis, which is also a typical sign of human scleroderma [54]. The literature describes how treatment with BLM in mice leads to thickening of both the epidermis and dermis [54, 67]. In the mice shown here, only epidermal thickening was observed, not dermal thickening, but with a significant thickening of the collagen fibers in the dermis. One possible reason for this could be that BLM treatment was only carried out for 11 days to assess differential susceptibility to fibrosis in an early stage of disease. There were no pronounced changes in Stabilin‐deficient mice, indicating that Stabilin deficiency in this model does not exert a strong influence on cutaneous fibrosis. Furthermore, we observed systemic effects of BLM treatment. It has been previously described that subcutaneous injection of BLM leads to lung fibrosis and changes in alveolar structure in mice, in addition to scleroderma [54, 55]. Evaluation of Sirius Red staining of the lungs in the mice from this study showed a slight, but not significant, increase in lung fibrosis levels in WT mice. However, a significant influence on the alveolar structure of the lungs was already evident. As described in the literature, all three genotypes exhibited a notable reduction in total alveolar area. Nevertheless, this reduction was significant only in WT mice. Based on these two results, it appears that the lungs of WT mice may react somewhat more strongly to subcutaneous injection of bleomycin compared to the lungs of Stabilin‐deficient mice.

In the fibrotic model of Bleomycin injection, IF staining of the skin showed only a slight increase in the TGFBi signal in WT Bleomycin‐treated mice and a significantly higher TGFBi level in *Stab1* ^−/−^ mice compared to *Stab2* ^−/−^ mice comparing treated mice only. BLM treatment had no effect on TGFBI skin staining in *Stab2* ^−/−^ mice. In plasma analysis, there was a significant reduction in TGFBi levels in *Stab1* ^−/−^ mice after BLM treatment.

The fact that BLM had no effect on local and circulation TGFBi in tissue and plasma in *Stab2* ^−/−^ mice may be attributed to the compensatory ability of Stab1 for the loss of Stab2 in this model. The decrease of TGFBi level in plasma of *Stab1* ^−/−^ mice could be explained by both enhanced local depositions of TGFBi in the tissue and an increased compensatory activity of Stab2.

The subcutaneous injection of bleomycin did not alter overall Postn levels, although hair follicles were lost. However, BLM treatment decreased plasma Postn levels compared to vehicle‐treated controls of the same genotype. In the literature, a significant increase in Postn expression in the skin and serum of SSc patients was demonstrated [68].

To conclude, we could show that the deficiency of Stab2 compared to deficiency for Stab1 seemed to exert a small protection in the inflammatory IMQ model. These results suggest that the inhibition of Stab2 in inflammatory skin diseases could potentially have a positive effect and may thus represent a therapeutic approach. In the fibrotic model induced by BLM Stabilin deficiency seems to have no grave influence on cutaneous fibrosis.

The inhibition of Stab1 is evaluated in a phase 1/2 clinical trial as adjuvant cancer immunotherapy [69]. Furthermore, we have already demonstrated that the inhibition of either Stab1 or Stab2 also has a protective effect on plaque development in mice in an atherosclerotic model [33].

The results of this study show that inhibiting either Stab1 or Stab2 does not appear to pose a strongly increased risk of exacerbating fibrotic or inflammatory skin conditions, which suggests that an anti‐Stabilin targeted therapy in atherosclerosis or cancer therapy might not lead to adverse effects in inflammatory or fibrotic skin conditions. Whether monoclonal‐antibody‐mediated inhibition of Stab2 may be useful as therapy for psoriasis needs to be evaluated in future studies.

## Conflict of interest

The authors declare no conflict of interest.

### Peer review

The peer review history for this article is available at https://www.webofscience.com/api/gateway/wos/peer‐review/10.1002/2211‐5463.13857.

## Author contributions

Conceptualization, CG and TL; methodology, CG and TL; software, CD, JK, TL and AR; validation, JK, TL and CG; formal analysis, CG and TL; investigation, CD, AR, TL and JK; resources, CG; data curation, JK, TL, and AR; writing—original draft preparation, JK and TL; writing—review and editing, CG; visualization, TL and JK; supervision, TL and CG; project administration, CG and TL; funding acquisition, CG. All authors have read and agreed to the published version of the manuscript.

## Supporting information


**Fig. S1.** Skin alteration after Imiquimod treatment.
**Fig. S2.** Single parameters of PASI score from Imiquimod‐treated mice.
**Fig. S3.** Histological analysis of mouse skin after Bleomycin treatment.
**Fig. S4.** Repeated subcutaneous injection of Bleomycin leads to an alteration in lung tissue.
**Fig. S5.** Repeated subcutaneous injection of Bleomycin has no effects on kidney tissue.
**Fig. S6.** Effects of subcutaneous injection of Bleomycin on stabilin ligands TGFBi and Postn in lung tissue.
**Fig. S7.** Effects of Imiquimod on stabilin ligand TGFBi in kidney tissue.
**Fig. S8.** Effects of Imiquimod on stabilin ligand Postn in kidney tissue.
**Fig. S9.** Effects of Bleomycin on stabilin ligands TGFBi and Postn in kidney.
**Fig. S10.** Effects of Imiquimod on stabilin ligand TGFBi in liver tissue.
**Fig. S11.** Effects of Imiquimod on stabilin ligand Postn in liver tissue.
**Fig. S12.** Effects of subcutaneous injection of Bleomycin on stabilin ligands TGFBi and Postn in liver tissue.
**Fig. S13.** Effects of Stabilin Ligands TGFBi and Postn in plasma of IMQ‐treated mice, comparison of sexes.
**Fig. S14.** Effects of subcutaneous injection of Bleomycin on stabilin ligands TGFBi and Postn in skin and plasma.

## Data Availability

The data that support the findings of this study are available from the corresponding authors, upon reasonable request.
