# Supplementary material for: Phenotypical and biochemical characterization of murine psoriasiform and fibrotic skin disease models in Stabilin‐deficient mice
Source: FEBS Open Bio. 2024 Jun 30;14(9):1455–70. doi: 10.1002/2211-5463.13857 (PMC11492309; doi:10.1002/2211-5463.13857)
Supplement: Supplementary file 1 — Fig. S1. Skin alteration after Imiquimod treatment. Fig. S2. Single parameters of PASI score from Imiquimod‐treated mice. Fig. S3. Histological analysis of mouse skin after Bleomycin treatment. Fig. S4. Repeated subcutaneous injection of Bleomycin leads to an alteration in lung tissue. Fig. S5. Repeated subcutaneous injection of Bleomycin has no effects on kidney tissue. Fig. S6. Effects of subcutaneous injection of Bleomycin on stabilin ligands TGFBi and Postn in lung tissue. Fig. S7. Effects of Imiquimod on stabilin ligand TGFBi in kidney tissue. Fig. S8. Effects of Imiquimod on stabilin ligand Postn in kidney tissue. Fig. S9. Effects of Bleomycin on stabilin ligands TGFBi and Postn in kidney. Fig. S10. Effects of Imiquimod on stabilin ligand TGFBi in liver tissue. Fig. S11. Effects of Imiquimod on stabilin ligand Postn in liver tissue. Fig. S12. Effects of subcutaneous injection of Bleomycin on stabilin ligands TGFBi and Postn in liver tissue. Fig. S13. Effects of Stabilin Ligands TGFBi and Postn in plasma of IMQ‐treated mice, comparison of sexes. Fig. S14. Effects of subcutaneous injection of Bleomycin on stabilin ligands TGFBi and Postn in skin and plasma. [file FEB4-14-1455-s001.pdf]

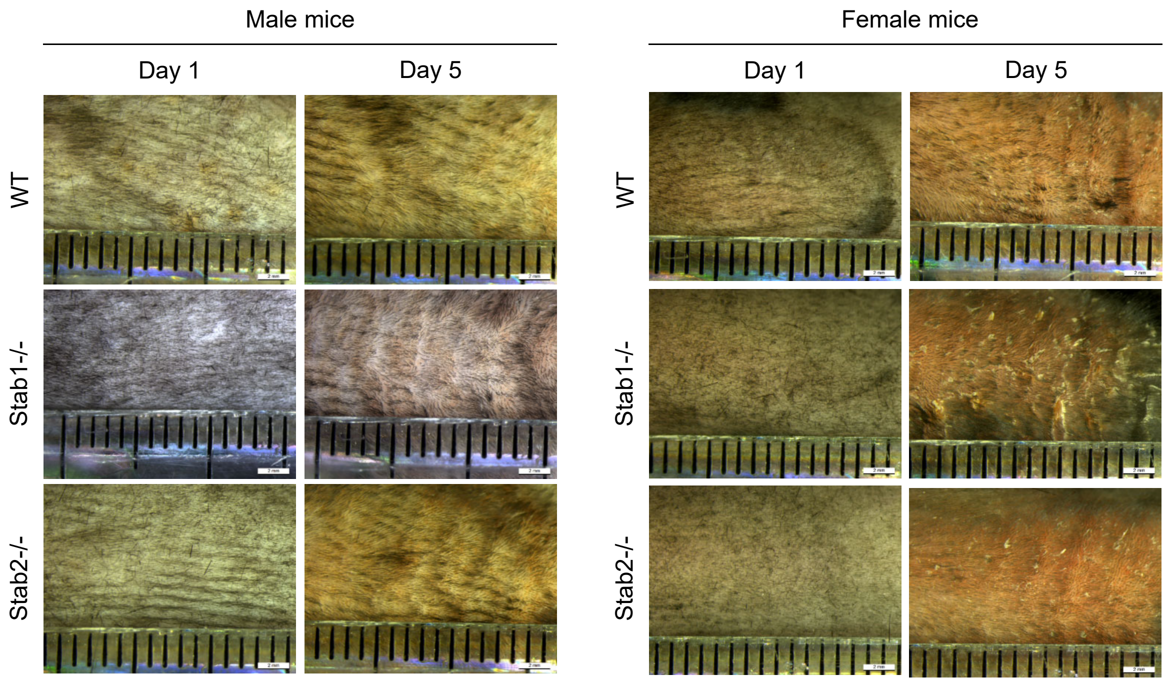

Suppl. Fig. 1 Skin alteration after Imiquimod treatment. Phenotypical presentation of male and female back skin after one and after five days of Imiquimod treatment. Scale bar = 2 mm.

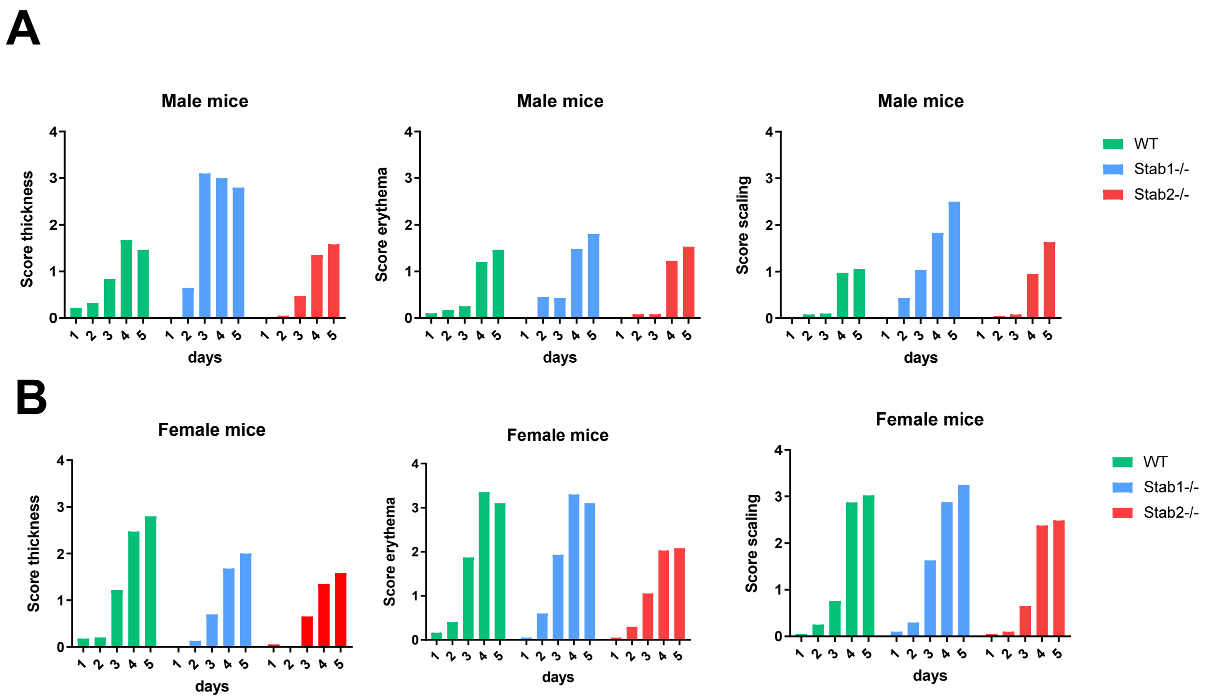

Suppl. Fig. 2 Single parameters of PASI score from Imiquimod treated mice. Single parameters of **(A)** male and **(B)** female mice (n=12 for WT mice, n=8 for *Stab1-/-* and *Stab2-/-* in each experimental condition and each gender).

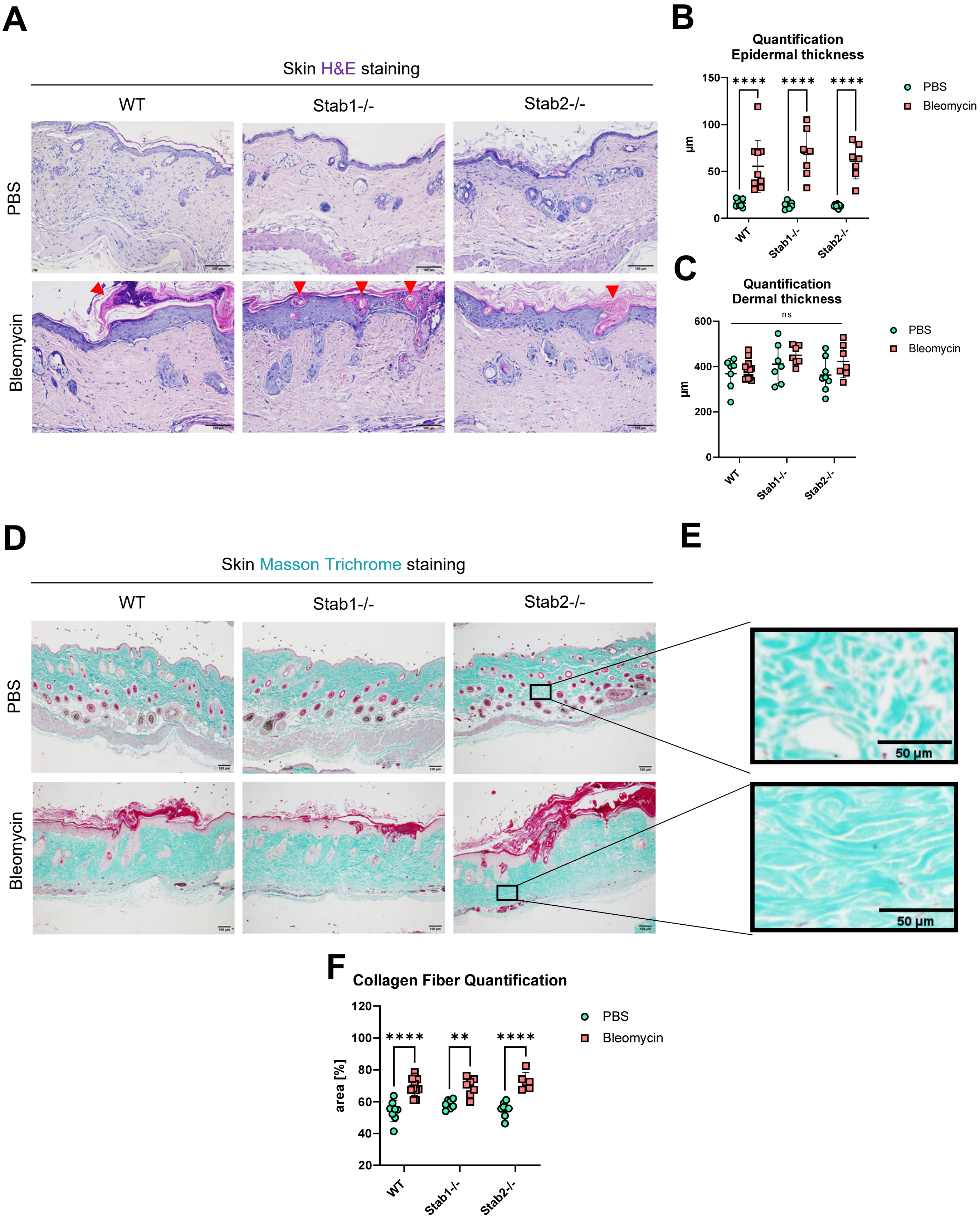

Suppl. Fig. 3 Histological analysis of mouse skin after Bleomycin treatment. (A) Representative skin sections of H&E staining after PBS and Bleomycin injection. Scale bar = 100 µm. **(B)** Quantification of epidermal thickness, quantified from H&E staining (n=8 per group). **(C)** Quantification of dermal thickness, quantified from H&E staining (n=8 per group). **(D)** Representative skin sections of Masson Trichrome staining after PBS and Bleomycin injection. Scale bar = 100 µm. **(E)** Enlarged areas of dermis of PBS and Bleomycin injected skin of *Stab2-/-* mice. **(F)** Quantification of collagen fibre density of PBS and Bleomycin injected mice, quantified from Masson Trichrome positive area (n=8 per group). Data of (B), (C) and (F) are presented as mean SD. The statistical analysis was performed with a two-way Anova. Symbols indicate significance: $p \leq 0.05$ = *; $p < 0.01$ = **; $p < 0.001$ = ***; $p < 0.0001$ = ****; n.s. = not significant.

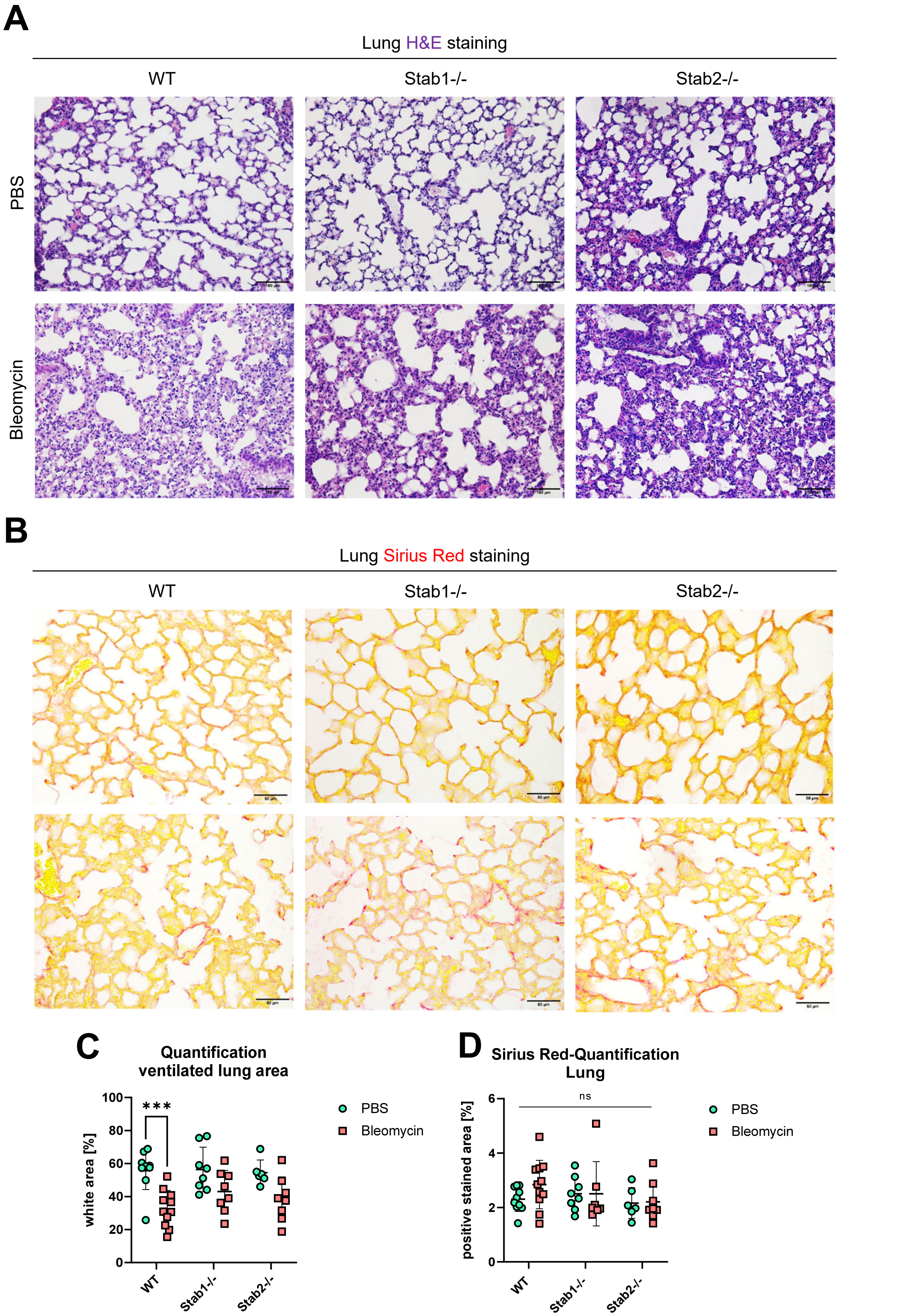

Suppl. Fig. 4 Repeated subcutaneous injection of Bleomycin leads to an alteration in lung tissue. (A) H&E staining of lung tissue. Scale bar = 100 µm. **(B)** Sirius red staining of lung tissue. Scale bar = 50 µm. **(C)** Quantification of ventilated lung area (white area, n=8 per group). **(D)** Quantification of Sirius red positive area (n=8 per group). Data of (C) and (D) are presented as mean SD. The statistical analysis was performed with a two-way Anova. Symbols indicate significance: $p \leq 0.05$ = *; $p < 0.01$ = **; $p < 0.001$ = ***; $p < 0.0001$ = ****; n.s. = not significant.

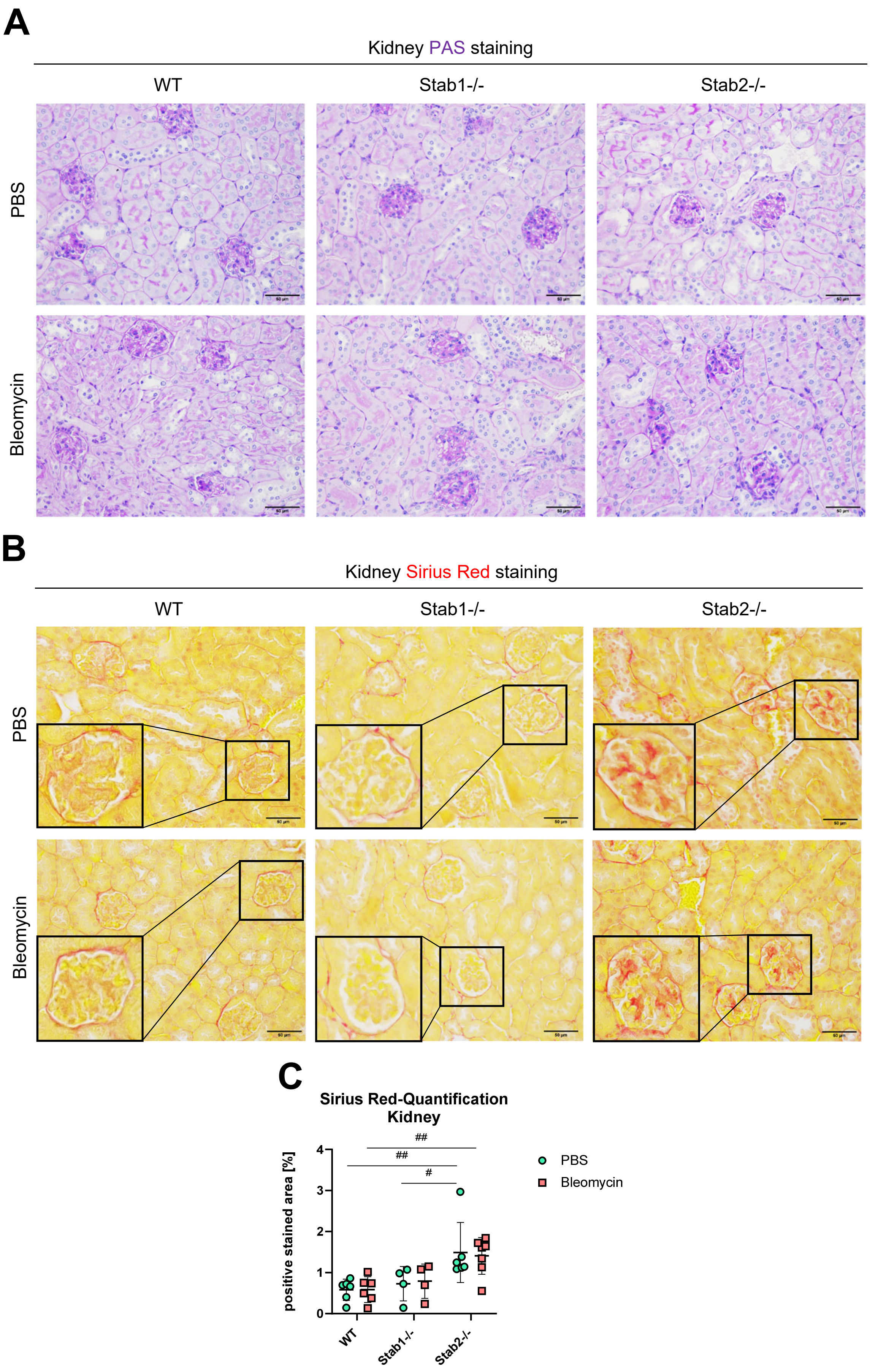

Suppl. Fig. 5 Repeated subcutaneous injection of Bleomycin has no effects on kidney tissue. (A) PAS staining of kidney tissue. Scale bar = 50 µm. **(B)** Sirius red staining of kidney tissue with enlarged glomeruli. Scale bar = 50 µm. **(C)** Quantification of Sirius red positive area (n≥4 per group). Data of (C) are presented as mean SD. The statistical analysis was performed with a two-way Anova. * show significances between treatments and # show significances between genotypes. Symbols indicate significances as follows: $p \leq 0.05$ = */#; $p < 0.01$ = **/##; $p < 0.001$ = ***/###; $p < 0.0001$ = ****/####; n.s. = not significant.

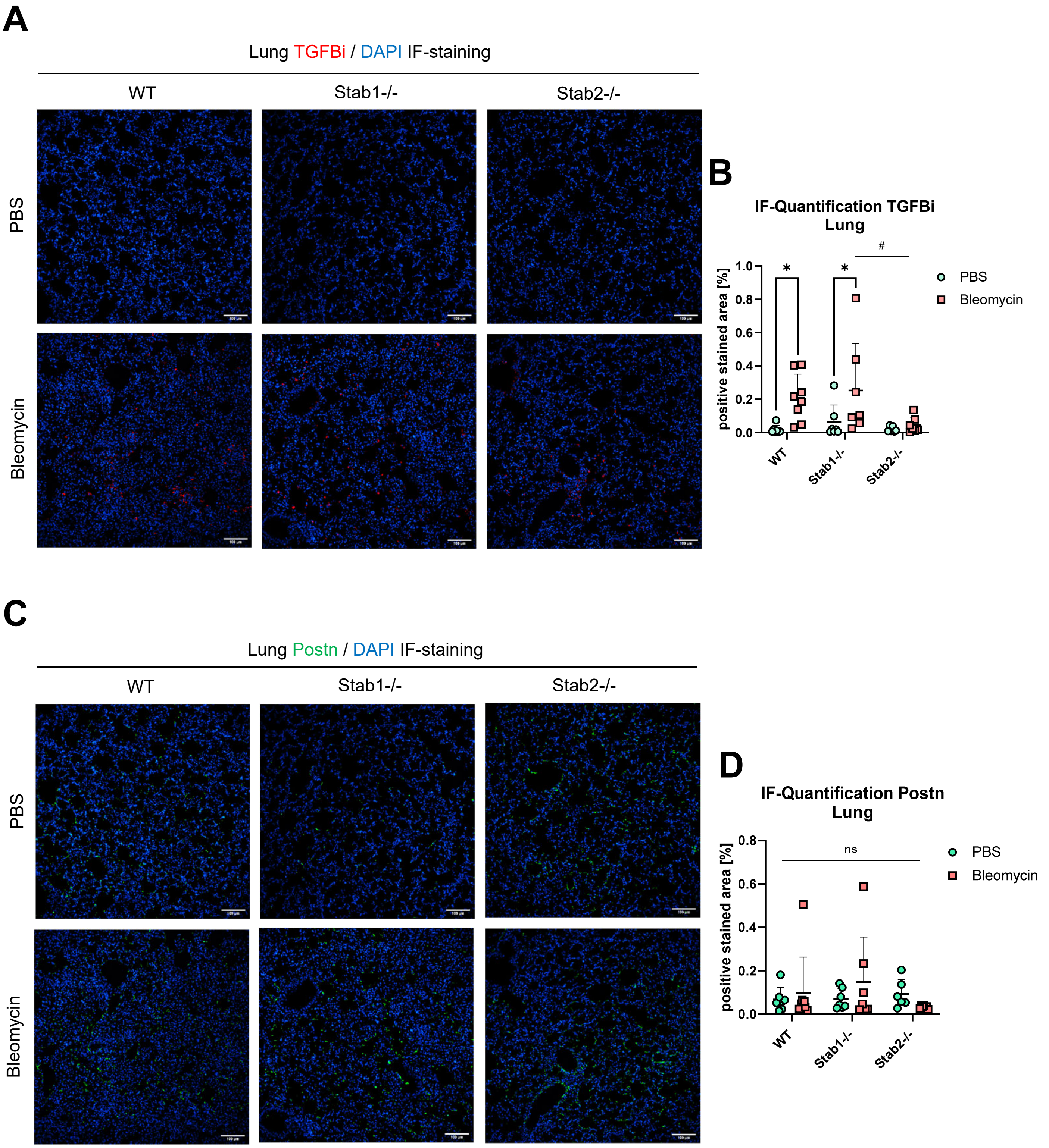

Suppl. Fig. 6 Effects of subcutaneous injection of Bleomycin on stabilin ligands TGFBi and Postn in lung tissue. (A) TGFBi IF-staining of representative lung sections. Scale bar = 100 µm. **(B)** Quantification of TGFBi IF-staining (n=8 per group). **(C)** Postn IF-staining of representative lung sections. Scale bar = 100 µm. **(D)** Quantification of Postn IF-staining (n=8 per group). Data of (B) and (D) are presented as mean SD. The statistical analysis was performed with a two-way Anova. * show significances between treatments and # show significances between genotypes. Symbols indicate significances as follows: $p \leq 0.05$ = */#; $p < 0.01$ = **/##; $p < 0.001$ = ***/###; $p < 0.0001$ = ****/####; n.s. = not significant.

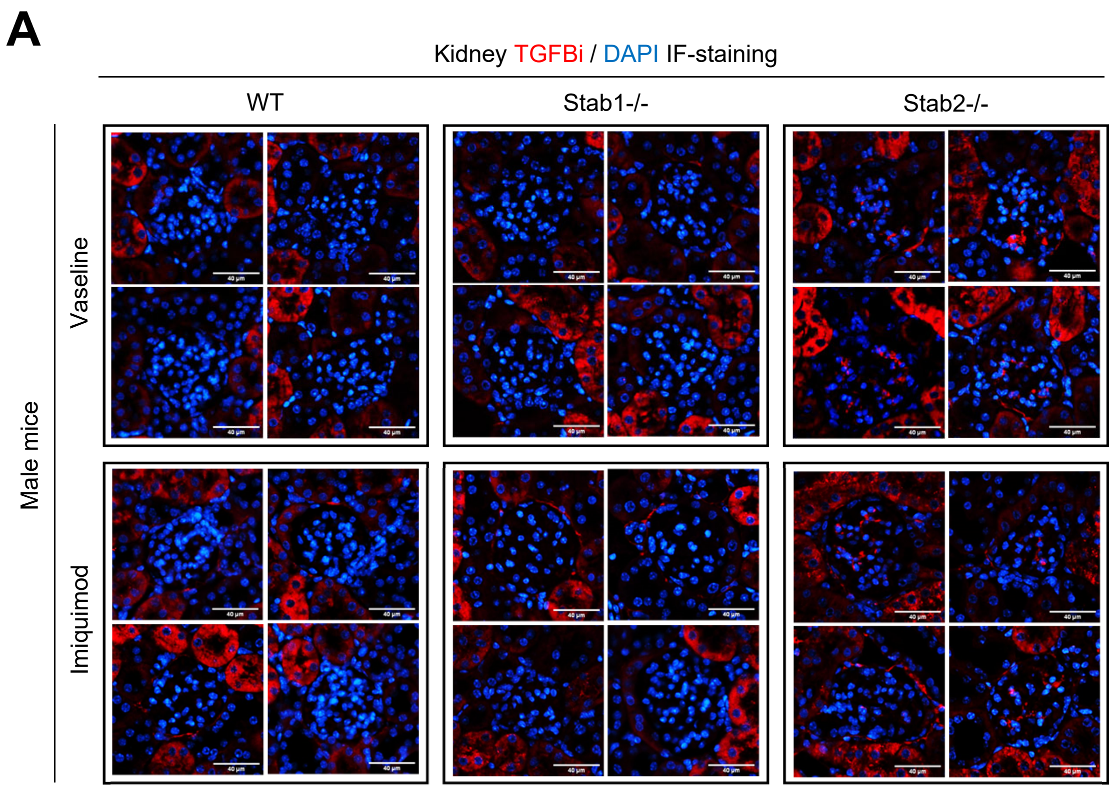

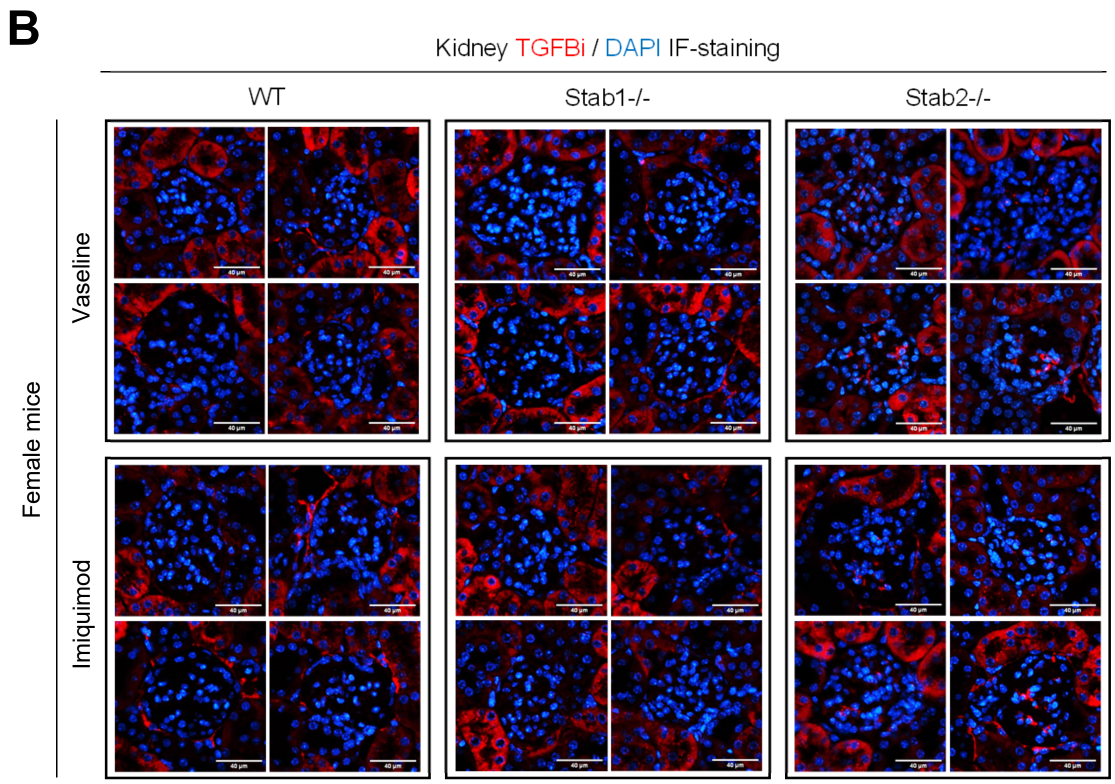

Suppl. Fig. 7 Effects of Imiquimod on stabilin ligand TGFBi in kidney tissue. IF-staining of TGFBi of glomeruli from representative kidney sections in **(A)** male mice and **(B)** female mice. Scale bar = 40 µm.
Per genotype and per treatment, four representative glomeruli of four different mice are shown.

A

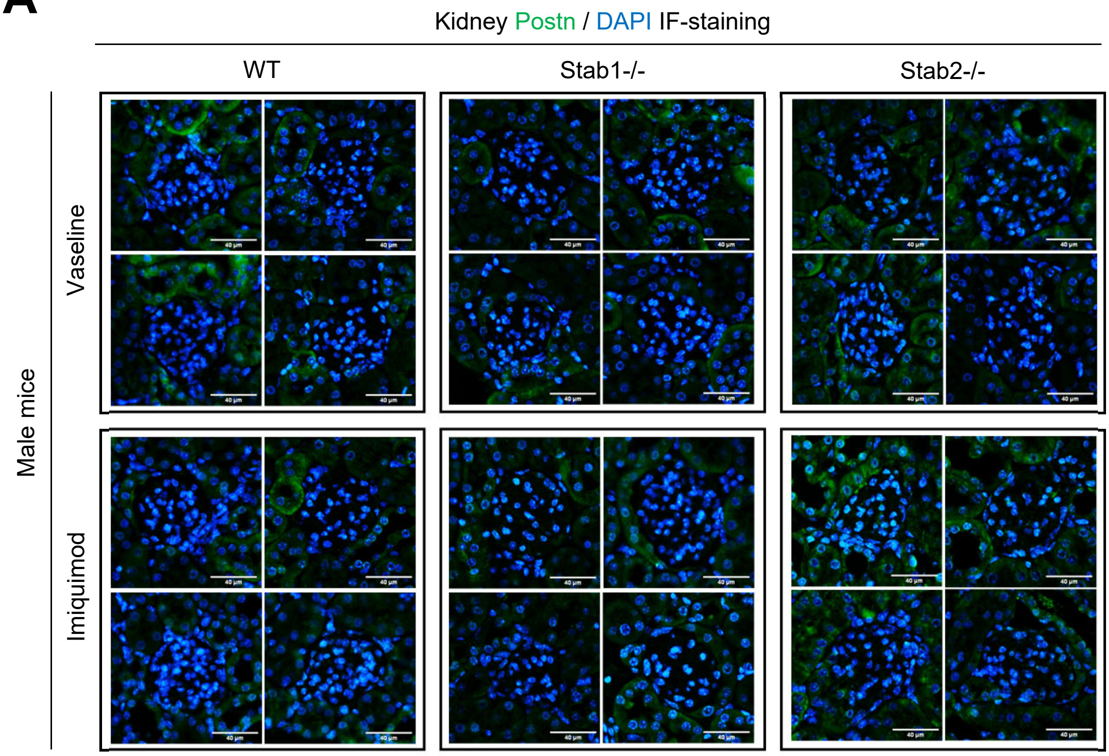

B

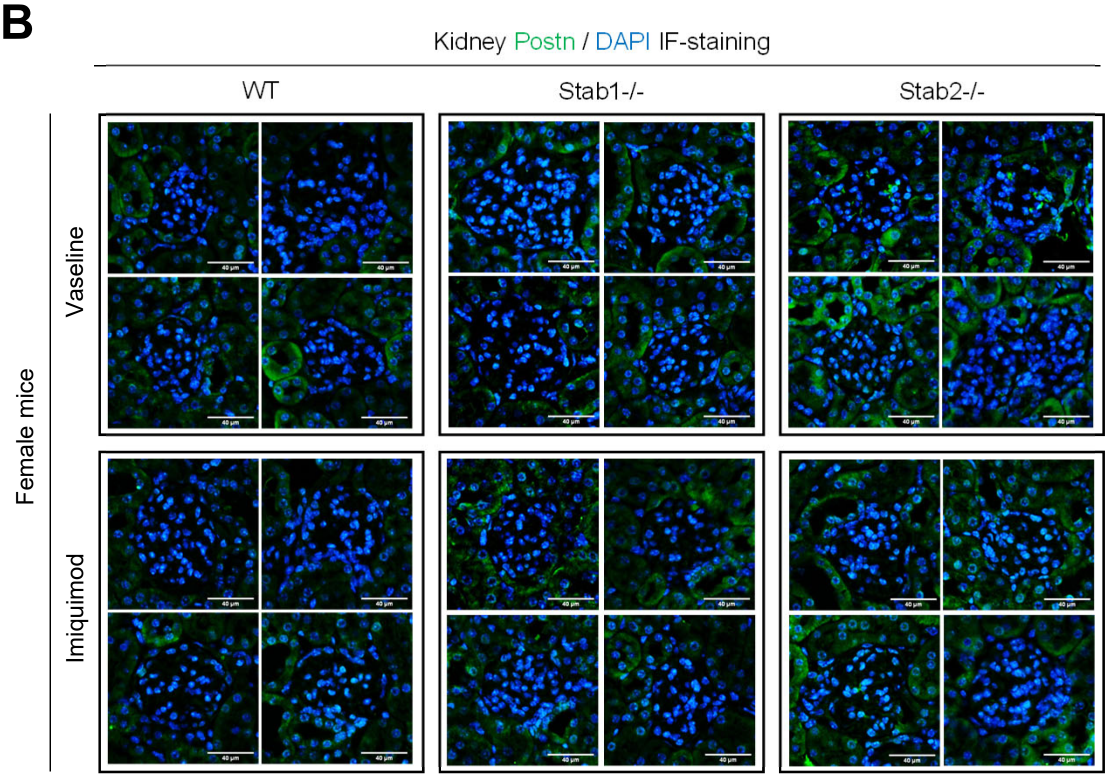

Suppl. Fig. 8 Effects of Imiquimod on stabilin ligand Postn in kidney tissue. IF-staining of Postn of glomeruli from representative kidney sections in **(A)** male mice and **(B)** female mice. Scale bar = 40 µm. Per genotype and per treatment, four representative glomeruli of four different mice are shown.

A

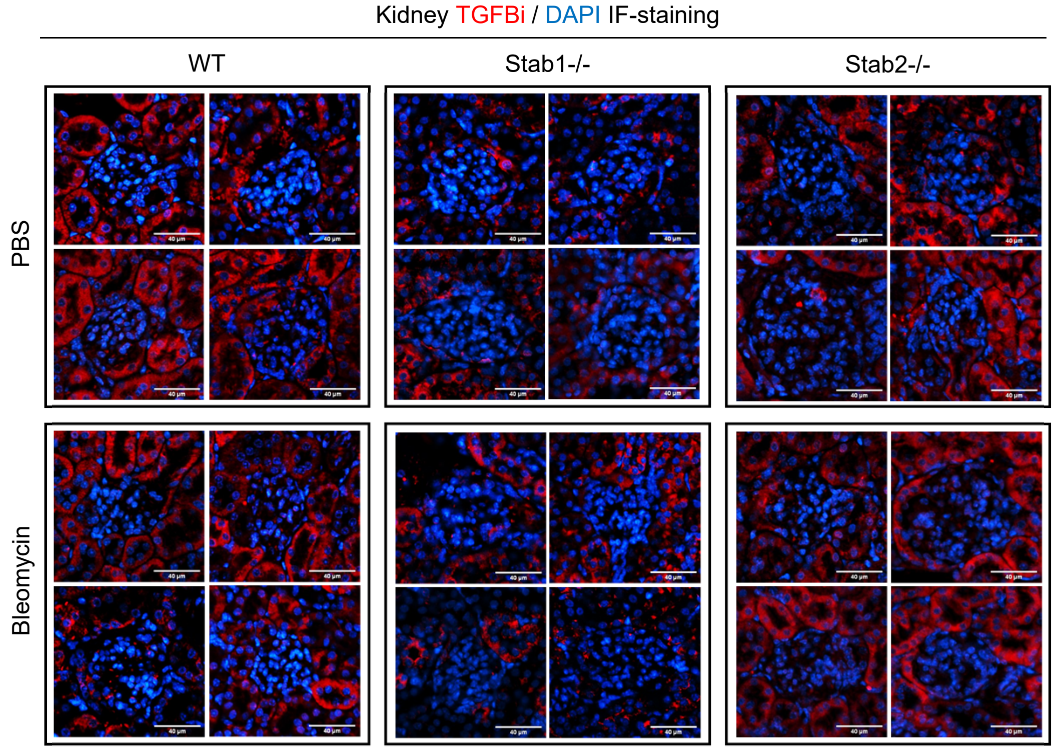

B

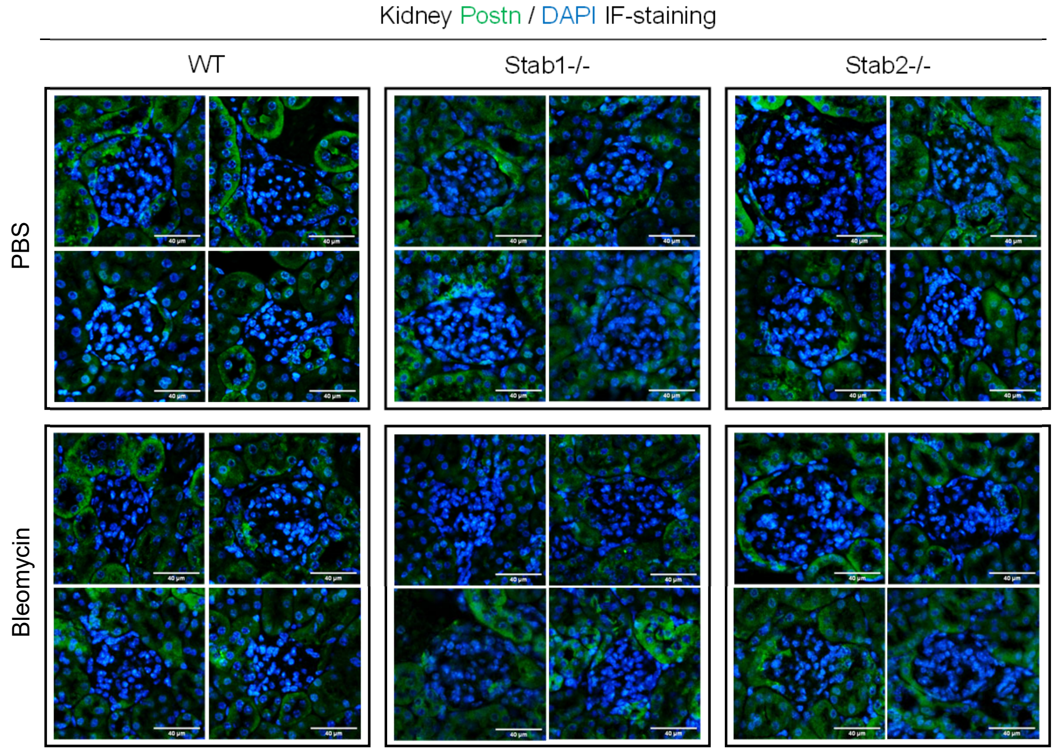

Suppl. Fig. 9 Effects of Bleomycin on stabilin ligands TGFBi and Postn in kidney. IF-staining of **(A)** TGFBi and **(B)** Postn of glomeruli from representative kidney sections. Scale bar = 40 µm.
Per genotype and per treatment, four glomeruli of four different mice are shown.

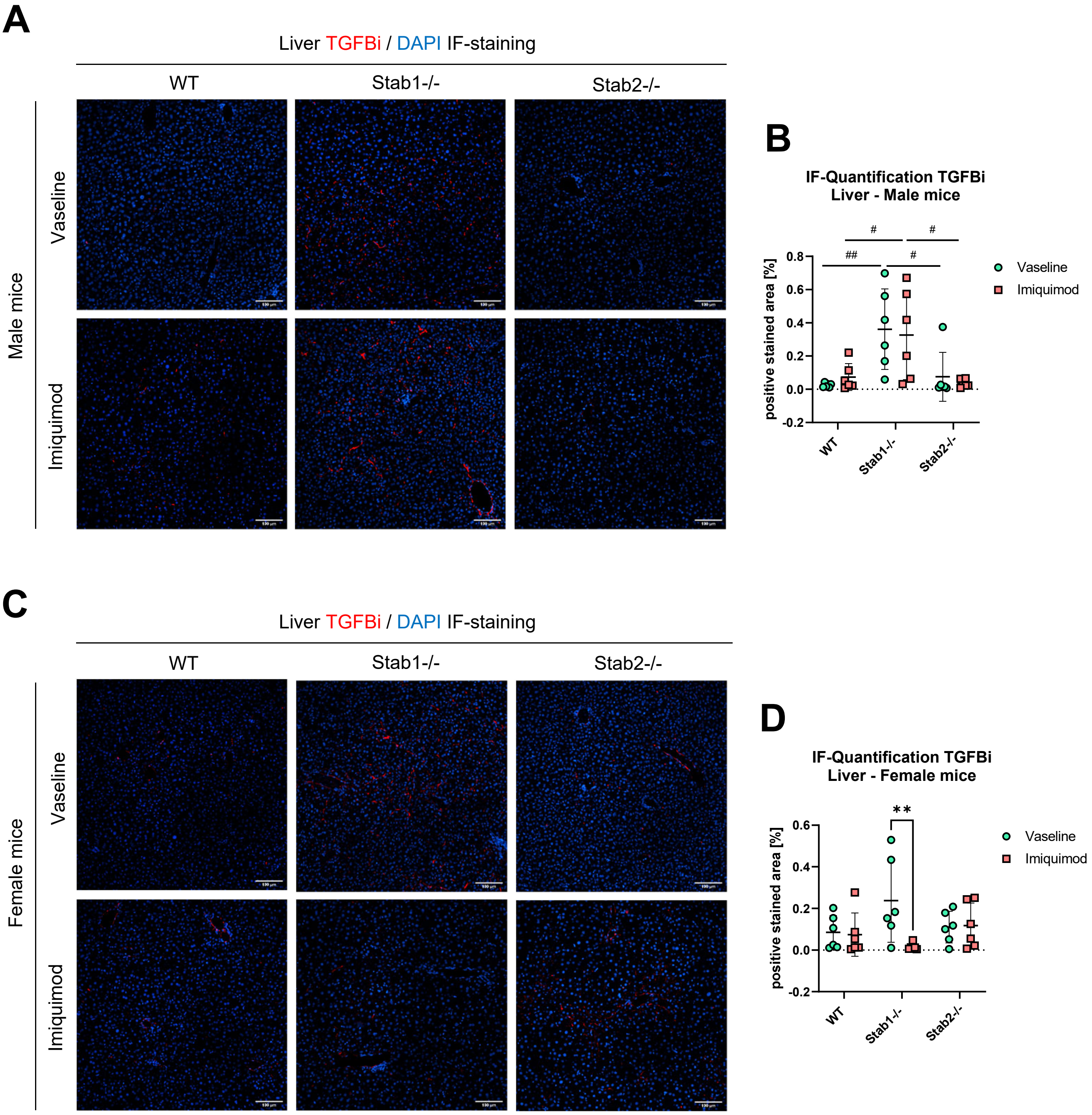

Suppl. Fig. 10 Effects of Imiquimod on stabilin ligand TGFBi in liver tissue. (A) IF-staining of TGFBi of representative liver sections in male mice. Scale bar = 100 µm. **(B)** Quantification of TGFBi IF-staining from male mice (n=6 per group). **(C)** IF-staining of TGFBi of representative liver sections in female mice. Scale bar = 100 µm. **(D)** Quantification of TGFBi IF-staining from female mice (n=6 per group). Data of (B) and (D) are presented as mean SD. The statistical analysis was performed with a two-way Anova. * show significances between treatments and # show significances between genotypes. Symbols indicate significances as follows: $p \leq 0.05$ = */#; $p < 0.01$ = **/##; $p < 0.001$ = ***/###; $p < 0.0001$ = ****/####; n.s. = not significant.

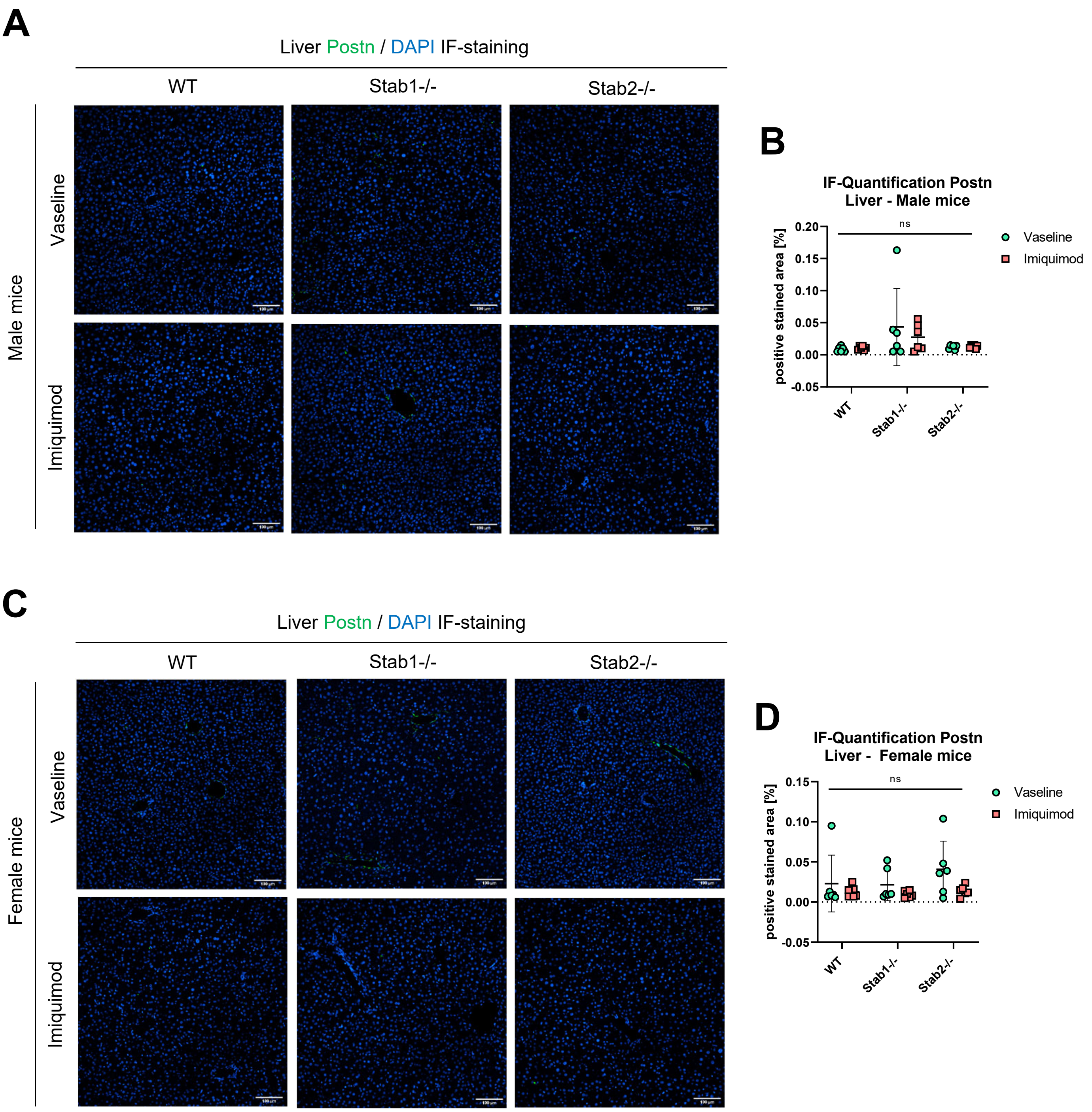

Suppl. Fig. 11 Effects of Imiquimod on stabilin ligand Postn in liver tissue. (A) IF-staining of Postn of representative liver sections in male mice. Scale bar = 100 µm. (B) Quantification of Postn IF-staining from male mice (n=6 per group). (C) IF-staining of Postn of representative liver sections in female mice. Scale bar is 100 µm. (D) Quantification of Postn IF-staining from female mice (n=6 per group). Data of (B) and (D) are presented as mean SD. The statistical analysis was performed with a two-way Anova. n.s. = not significant.

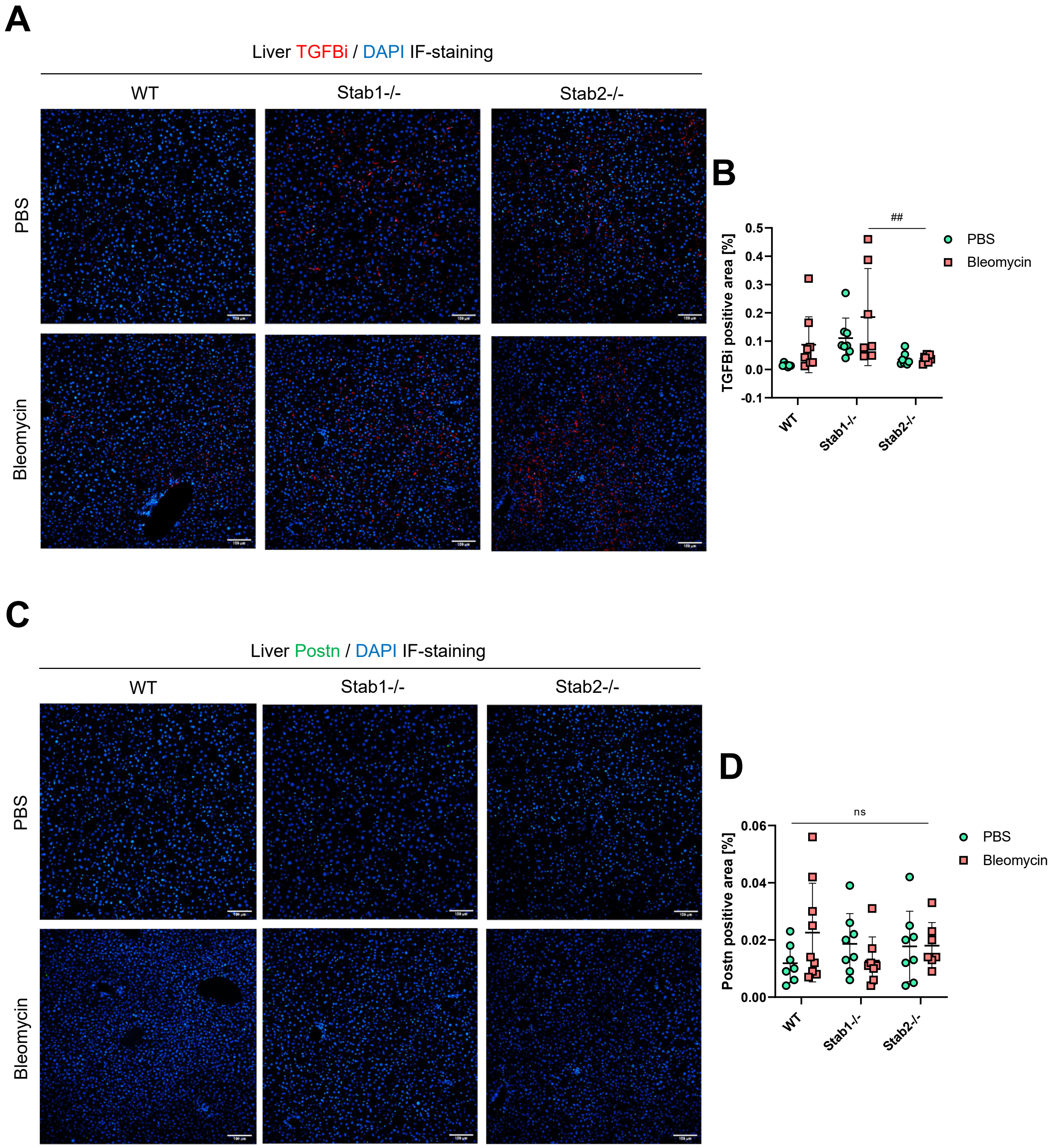

Suppl. Fig. 12 Effects of subcutaneous injection of Bleomycin on stabilin ligands TGFBi and Postn in liver tissue. (A) TGFBi IF-staining of representative liver sections. Scale bar = 100 µm. **(B)** Quantification of TGFBi IF-staining (n=6 per group). **(C)** Postn IF-staining of representative liver sections. Scale bar = 100 µm. **(D)** Quantification of Postn IF-staining (n=6 per group). Data of (B) and (D) are presented as mean SD.The statistical analysis was performed with a two-way Anova. * show significances between treatments and # show significances between genotypes. Symbols indicate significances as follows: p ≤ 0.05 = */#; p < 0.01 = **/##; p < 0.001 = ***/###; p < 0.0001 = ****/####; n.s. = not significant.

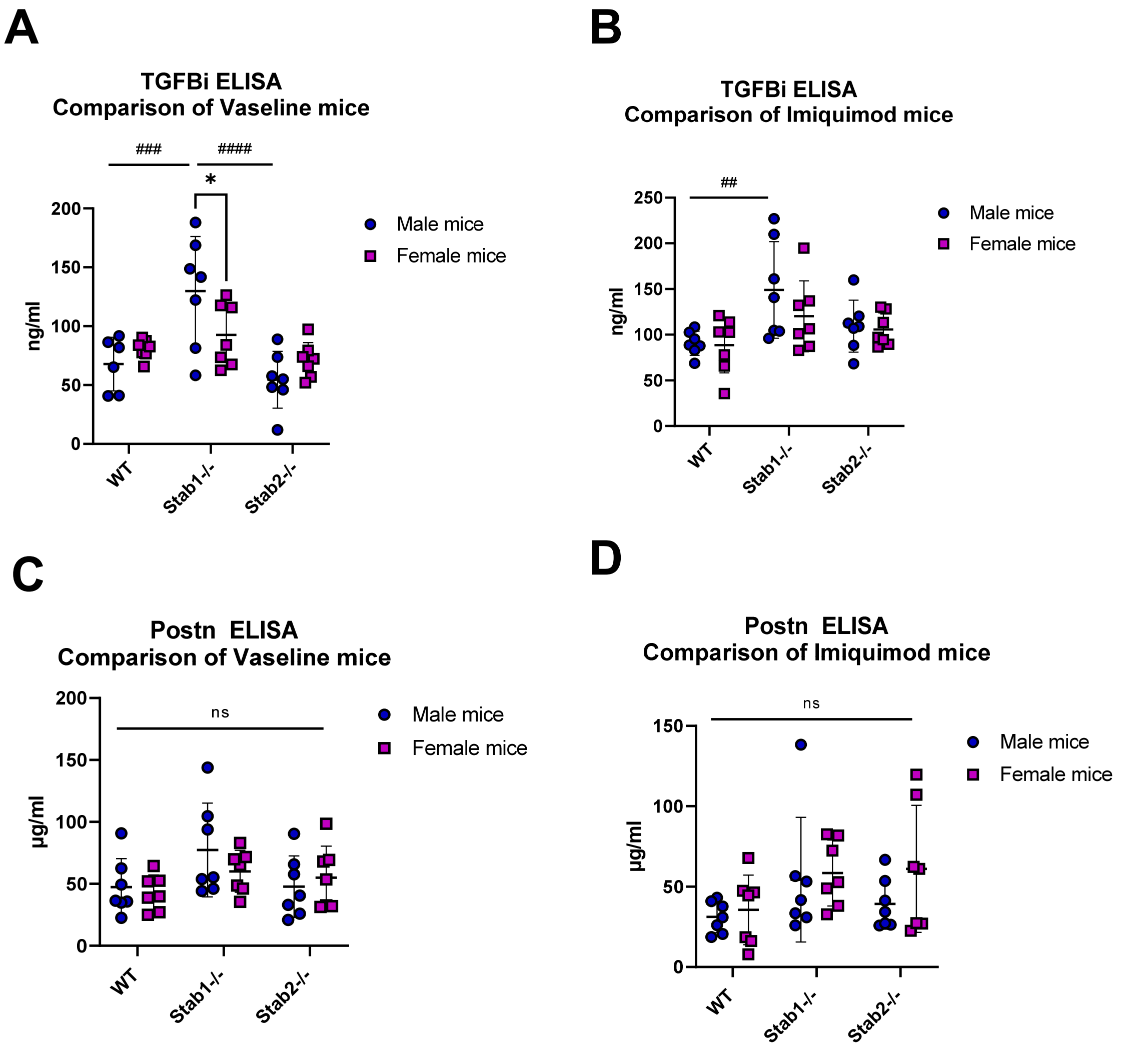

Suppl. Fig. 13 Effects of Stabilin Ligands TGFBi and Postn in plasma of IMQ treated mice, comparison of sexes. (A) Comparison of TGFBi level in plasma of male and female mice, treated with Vaseline. **(B)** Comparison of TGFBi level in plasma of male and female mice, treated with Imiquimod. **(C)** Comparison of Postn level in plasma of male and female mice, treated with Vaseline. **(D)** Comparison of Postn level in plasma of male and female mice, treated with Imiquimod. Data of (A), (B), (C) and (D) are presented as mean SD. The statistical analysis was performed with a two-way Anova. * show significances between genders and # show significances between genotypes. Symbols indicate significances as follows: $p \leq 0.05$ = */#; $p < 0.01$ = **/##; $p < 0.001$ = ***/###; $p < 0.0001$ = ****/####; n.s. = not significant.

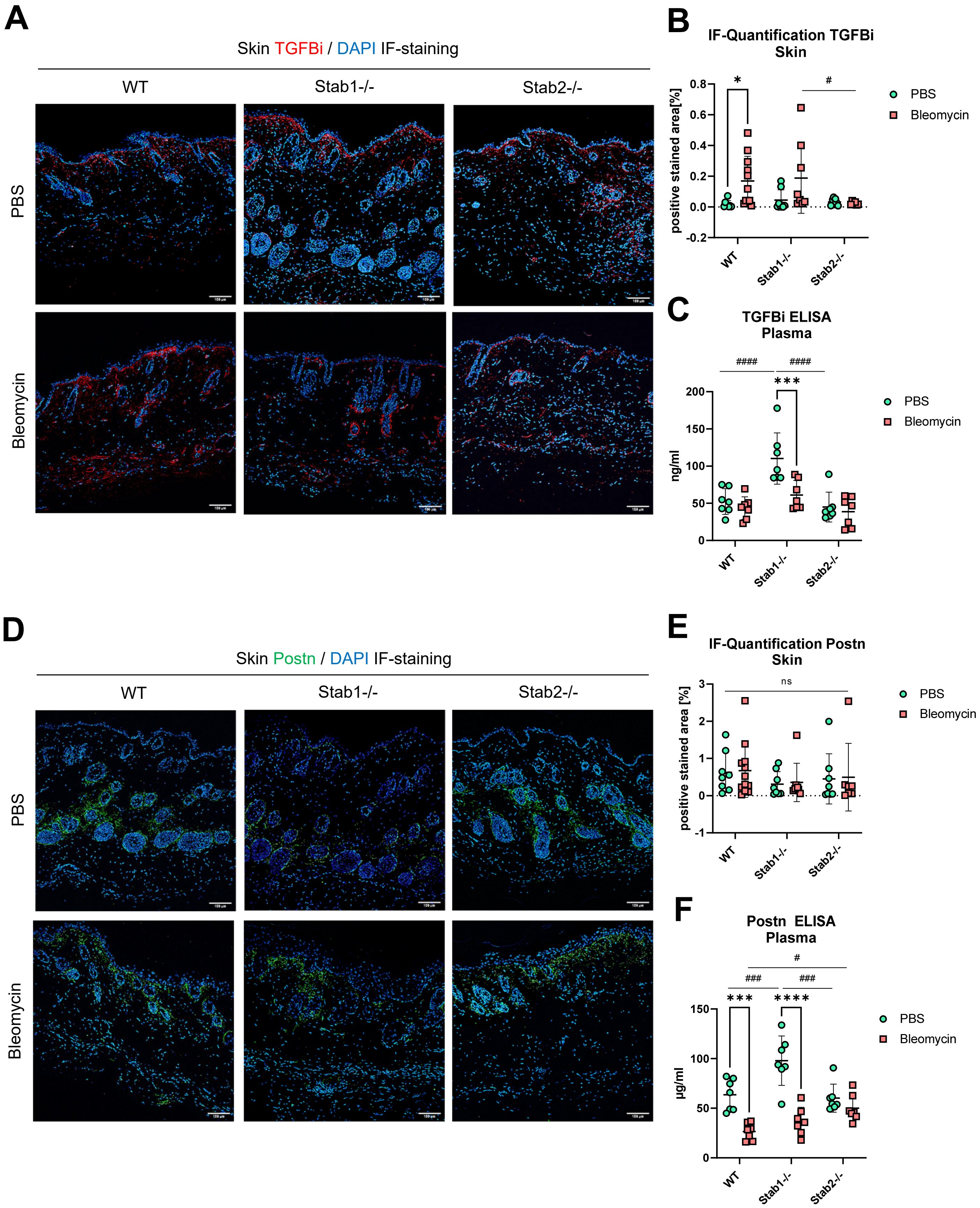

Suppl. Fig. 14 Effects of subcutaneous injection of Bleomycin on stabilin ligands TGFBi and Postn in skin and plasma. (A) TGFBi IF-staining of representative skin sections. Scale bar is 100 µm. **(B)** Quantification of TGFBi IF-staining (n≥6 per group). **(C)** Plasma POSTN levels (ELISA; n≥6 per group).**(D)** Postn IF-staining of representative skin sections. Scale bar is 100 µm. **(E)** Quantification of Postn IF-staining. **(F)** Plasma POSTN levels (ELISA; n≥6 per group). Data of (B), (C), (E) and (F) are presented as mean SD. The statistical analysis was performed with a two-way Anova. * show significances between treatments and # show significances between genotypes. Symbols indicate significances as follows: p ≤ 0.05 = */#; p < 0.01 = **/##; p < 0.001 = ***/###; p < 0.0001 = ****/####; n.s. = not significant.
